# Deficiency of neutral cholesterol ester hydrolase 1 (NCEH1) impairs endothelial function in diet-induced diabetic mice

**DOI:** 10.1186/s12933-024-02239-6

**Published:** 2024-04-25

**Authors:** Hai-Jian Sun, Zhang-Rong Ni, Yao Liu, Xiao Fu, Shi-Yi Liu, Jin-Yi Hu, Qing-Yi Sun, Yu-Chao Li, Xiao-Hui Hou, Ji-Ru Zhang, Xue-Xue Zhu, Qing-Bo Lu

**Affiliations:** 1 Department of Physiology, Wuxi School of Medicine, Jiangnan University, Wuxi, 214122 China; 2 State Key Laboratory of Natural Medicines, China Pharmaceutical University, No. 24 Tongjia Lane, Nanjing, 210009 China; 3 Department of Cardiac Ultrasound, The Fourth Affiliated Hospital of Nanjing Medical University, Nanjing, 210000 Jiangsu China; 4 Department of Endocrinology, Affiliated Hospital of Jiangnan University, Jiangnan University, Wuxi, 214125 China; 5 Department of Basic Medicine, Wuxi School of Medicine, Jiangnan University, Wuxi, 214122 China

**Keywords:** Diabetes, Endothelial dysfunction, Nitric oxide, NCEH1, eNOS

## Abstract

**Background:**

Neutral cholesterol ester hydrolase 1 (NCEH1) plays a critical role in the regulation of cholesterol ester metabolism. Deficiency of NCHE1 accelerated atherosclerotic lesion formation in mice. Nonetheless, the role of NCEH1 in endothelial dysfunction associated with diabetes has not been explored. The present study sought to investigate whether NCEH1 improved endothelial function in diabetes, and the underlying mechanisms were explored.

**Methods:**

The expression and activity of NCEH1 were determined in obese mice with high-fat diet (HFD) feeding, high glucose (HG)-induced mouse aortae or primary endothelial cells (ECs). Endothelium-dependent relaxation (EDR) in aortae response to acetylcholine (Ach) was measured.

**Results:**

Results showed that the expression and activity of NCEH1 were lower in HFD-induced mouse aortae, HG-exposed mouse aortae ex vivo, and HG-incubated primary ECs. HG exposure reduced EDR in mouse aortae, which was exaggerated by endothelial-specific deficiency of NCEH1, whereas NCEH1 overexpression restored the impaired EDR. Similar results were observed in HFD mice. Mechanically, NCEH1 ameliorated the disrupted EDR by dissociating endothelial nitric oxide synthase (eNOS) from caveolin-1 (Cav-1), leading to eNOS activation and nitric oxide (NO) release. Moreover, interaction of NCEH1 with the E3 ubiquitin-protein ligase ZNRF1 led to the degradation of Cav-1 through the ubiquitination pathway. Silencing Cav-1 and upregulating ZNRF1 were sufficient to improve EDR of diabetic aortas, while overexpression of Cav-1 and downregulation of ZNRF1 abolished the effects of NCEH1 on endothelial function in diabetes. Thus, NCEH1 preserves endothelial function through increasing NO bioavailability secondary to the disruption of the Cav-1/eNOS complex in the endothelium of diabetic mice, depending on ZNRF1-induced ubiquitination of Cav-1.

**Conclusions:**

NCEH1 may be a promising candidate for the prevention and treatment of vascular complications of diabetes.

**Supplementary Information:**

The online version contains supplementary material available at 10.1186/s12933-024-02239-6.

## Background

Diabetes has become a serious public health issue associated with increasing incidence around the world [1, 2]. Patients with diabetes have an elevated risk of cardiovascular events compared to age matched non-diabetic individuals [3, 4]. Cardiovascular complications are considered the primary causes of death and disability among diabetic patients [5, 6]. While the mechanisms behind the increased risk of cardiovascular ailments in diabetes are not fully understood, endothelial cell dysfunction plays a significant role in the development of atherosclerosis and related complications [7, 8]. Endothelial dysfunction manifests reduced nitric oxide (NO) bioavailability, increased endothelial inflammation, and uncontrolled oxidative stress [9]. Among which, reduced NO bioavailability is one the earliest pathologic events in various cardiovascular diseases, including diabetic vascular complications [10]. Clinical and animal studies have demonstrated that a high-fat diet (HFD) impairs endothelial function [11–14]. Chronic hyperglycemia state inactivated endothelial nitric oxide synthase (eNOS), reduced NO production and induces ROS generation in endothelial cells (ECs) [15], contributing to the pathogenesis of cardiovascular diseases.

Caveolin-1 (Cav-1) is a main coat protein of caveolae that is abundantly expressed in ECs [16]. The activity of eNOS is tightly regulated by its interaction with Cav-1 in which the dissociation of eNOS from Cav-1 increases the enzyme function of eNOS [17]. Enhanced NO-dependent vascular function were observed in blood vessels from Cav-1 knockout mice [18, 19], indicating that Cav-1 scaffolding domain are responsible for inhibiting eNOS-derived NO release in ECs. A mutant cell-permeable scaffolding domain peptide called Cavnoxin enhances eNOS-derived NO synthesis and vasodilation in mice [20]. Blocking the binding of Cav-1 to eNOS provides atheroprotection in diabetes-accelerated atherosclerosis [21]. These findings indicate a therapeutic application for regulating the eNOS/Cav-1 interaction in cardiovascular and metabolic diseases, including diabetes.

Cholesteryl ester (CE) is a storage form of lipids, and the metabolism of CE in cells includes two reverse biological processes [22]. One process is the synthesis of CE, in which free cholesterol and free fatty acids are mainly esterified to CE [23]. The other process is the hydrolysis of CE, which is mainly hydrolyzed into free cholesterol and free fat under the catalysis of neutral cholesteryl ester hydrolase 1 (NCEH1) and Lysosome acid lipase (LAL) [24]. NCEH1 hydrolyzes CE in cytoplasm, and CE hydrolyzed by NCEH1 is removed from foam cells into high-density lipoprotein (HDL) [25]. It has been reported that the activity of NCEH1 tends to be lower in macrophage-derived foam cells and macrophages from atherosclerosis-prone C57BL/6J mice [26, 27]. Ablation of NCEH1 promotes foam cell formation and accelerates atherosclerotic lesion formation in atherosclerosis-prone mice [28]. Thus, the impaired NCEH1 activity may result in the progression of atherosclerosis. However, whether malfunction of NCEH1 is pathologically important in diabetic cardiovascular complications has yet to be fully elucidated, and whether restored NCEH1 activity effectively protected endothelial function in diabetes remains undefined. Therefore, the aim of the present study is to clarify whether NCEH1 protects against endothelial dysfunction induced by high-fat diet (HFD), and if so, to explore the underlying molecular mechanisms involved.

## Methods

### Chemicals and reagent

Primary antibodies against NCEH1 (14021-1-AP), β-actin (81115-1-RR), HRP-conjugated Affinipure Goat Anti-Rabbit IgG(H + L) (SA00001-2), HRP-conjugated Affinipure Goat Anti-Mouse IgG(H + L) (SA00001-1) and CD31 (66065-2-Ig) were purchased from Proteintech Group (Rosemont, IL, USA). Griess Reagent (G2930) was obtained from Promega (Madison, WI, USA). ZNRF1 antibody (orb1224) was purchased from Biorbyt (Cambridge, United Kingdom). 4-amino-5-methylamino-2’,7’-difluorofluorescein diacetate (DAF-FM diacetate, D23844), Goat anti-Rabbit IgG (H + L) Highly Cross-Adsorbed Secondary Antibody, Alexa Fluor™ 594 (A-11,037) and Goat anti-Mouse IgG (H + L) Highly Cross-Adsorbed Secondary Antibody, Alexa Fluor™ 488 (A-11,029) were procured from Thermo Fisher Scientific (Carlsbad, CA, USA). The commercial kits for measurement of triglyceride (TG, A110-1-1), insulin (H203-1-2), and total cholesterol (TC, A111-1-1) were purchased from Jiancheng Bioengineering Institute (Nanjing, China). Acetylcholine (Ach, PHR1546), sodium nitroprusside (SNP, BP453), phenylephrine (Phe, P1240000), chloroquine (C6628), cycloheximide (CHX, 66-81-9), D-glucose (PHR1000), and 4’,6-Diamidine-2’-phenylindole dihydrochloride (DAPI) were purchased from Sigma (St. Louis, USA). Primary antibodies against caveolin-1 (sc-53,564), eNOS (sc-376,751), Protein A/G PLUS-Agarose (sc-2003) and primary Ub antibody (sc-8017) were purchased from Santa Cruz (CA, USA). Anti-eNOS (phospho S1177) antibody was procured from Abcam (Cambridge, MA, USA). The concentrations of chloroquine, CHX and MG-132 were selected according to previous reports [29, 30].

### Animals

All animal experiments were confirmed and approved by the Institutional Animal Care and Use Committee of the China Pharmaceutical University and Jiangnan University (approval license number, 202101016). The animal procedures were conformed to the Guide for the Care and Use of Laboratory Animals (NIH publication, 8th edition, 2011). All mice were purchased from Sibeifu (Beijing) Biotechnology Co., Ltd and were housed in a specific pathogen-free microisolator cages, with free access to autoclaved food and reverse-osmosis water, and the animals were caged under a controlled temperature and humidity room on a 12-h light/dark cycle. To minimize the environmental differences, all mice were maintained at least 7 days before experiments. C57BL/6 male mice at 8 weeks of age were used to induce obesity under a HFD (60% kcal as fat, Research Diets, New Brunswick, NJ, USA) for 12 weeks as previously reported [30–32]. Age-matched mice were fed with a standard chow for 12 weeks. For ex vivo experiments, the adult 8-week-old C57BL/6 mice were anesthetized, and intravenous injection of AAV5-TIE1-NCEH1 shRNA (AAV5.Tie.-P2A-NCEH1.WPREs.SV40pA, Forward, 5’-CATGATGCTTGTTCTGAGA-3’; Reverse, 5’-TCTCAGAACAAGCATCATG-3’), AAV5-TIE1-NCEH1 overexpression plasmids (AAV5.Tie.NCEH1-3xflag.WPREs.SV40pA), AAV5-TIE1-Cav-1 shRNA (AAV5.Tie.-P2A-Cav1.WPREs.SV40pA, Forward, 5’-GCAACATCTACAAGCCCAA; Reverse, 5’-TTGGGCTTGTAGATGTTGC-3’) [33], AAV5-TIE1-Cav-1 overexpression plasmids (AAV5.Tie.Cav-1-3xflag.WPREs.SV40pA), AAV5-TIE1-ZNRF1 shRNA (AAV5.Tie.-P2A-ZNRF1.WPREs.SV40pA, Forward, 5’-GCCTGTGCATCTATCACAA-3’; Reverse, 5’-TTGTGATAGATGCACAGGC-3’), AAV5-TIE1-ZNRF1 overexpression plasmids (AAV5.Tie.ZNRF1-3xflag.WPREs.SV40pA), or negative control AAV5 vectors (10^11^ viral genome particles for each mouse) was conducted as previously [34–36]. Under the control of a TIE1 promoter (pRP.ExSi-Tie2-RTTA), these AAV5 vector (Paizhen Biotechnology, China) injections led to the endothelial knockdown/overexpression of NCEH1, Cav-1 and ZNRF1 since the AAV5 constructs contained the binding sequence for the endothelial-specific promoter TIE1 [36, 37]. After AAV injections for 6 weeks, the mouse aortae were isolated and incubated for normal glucose (NG) or high glucose (HG) conditions. For in vivo experiments, HFD mice were subjected to intravenous injection of AAV5-TIE1-NCEH1 shRNA, AAV5-TIE1-NCEH1 overexpression plasmids (10^11^ viral genome particles for each mouse) after HFD feeding for 6 weeks. Six weeks later, the mouse aortae were collected for functional or molecular experiments. For euthanasia, animals were anesthetized with 5% isoflurane, anaesthesia was confirmed via tail pinch, and then sacrificed by cervical dislocation before blood vessel tissue removal.

### Organ culture of aortic rings

The aortic rings (2 mm in length) in mice were dissected in sterile PBS, and incubated with Dulbecco’s Modified Eagle’s Media (DMEM, Gibco, Gaithersberg, MD, USA) containing 10% fetal bovine serum (FBS), penicillin (100 IU/mL) and streptomycin (100 µg/mL). The mannitol (25 mM) was added as the NG osmotic control, whereas the addition of D-glucose (25 mM) was used as the HG model group. After incubation for 48 h, aortic rings were moved to a chamber filled with fresh physiological salt solution (PSS, NaCl 118 mM, KCl 3.4 mM, CaCl_2_ 2.5 mM, KH_2_PO_4_ 1.2 mM, MgSO_4_ 1.2 mM, NaHCO_3_ 25 mM, glucose 11.1 mM) solution and mounted in a myograph for the measurement of vascular tone [38].

### Evaluation of vasorelaxation

After sacrifice, the thoracic aortae of mouse were removed in oxygenated ice-cold physiological saline solution (PSS) and the thoracic aortae were cut into 4 rings of 1 mm in length. Changes in isometric tone of aortic rings were recorded by mounting onto two stainless steel wires in wire myograph (Model 620 M, Danish Myo Technology, Aarhus, Denmark), filling with 5 ml PSS aerated with 95% O_2_ and 5% CO_2_ at 37 °C. The arterial segments were stretched to an optimal baseline tension (3 mN) for 1 h before the experiments were started. After equilibration, the arterial rings were pre-contracted with phenylephrine (Phe, 1 µM) and rinsed in PSS. Endothelium dependent relaxation (EDR) was assessed by testing concentration-responses to cumulative concentrations of ACh (10^− 9^ to 10^− 4^ M) in Phe pre-contracted rings. Endothelium-independent relaxation to SNP (10^− 9^ to 10^− 5^ M) was carried out in endothelium-removed rings by gently rubbing with fine forceps. Relaxation at each concentration was expressed as the percentage of force in response to Phe.

### Double immunofluorescence staining

The arterial segments were cut into 5-µm sections and then dewaxed in xylene 3 times, and hydrated in alcohol. The sections were rinsed with PBS buffer and distilled water, and subjected to heat-repaired antigen for 10 min. After blocking with 1% BSA and 0.2% Triton-X for 5 min, the sections were incubated with NCEH1 anti-rabbit antibody and CD31 anti-mouse antibody at 4 °C overnight. Next, the sections were probed by Goat anti-Rabbit IgG connected with Alexa Fluor-594 and Goat anti-mouse IgG connected with Alexa Fluor-488 for 1 h at room temperature, followed by treatment with DAPI staining solution for 10 min. The immunofluorescence signals were captured by a fluorescence microscope (80i, Nikon, Tokyo, Japan).

### Measurement of NO

After the treatment, the aortic segments were incubated with DAF-FM diacetate (2 µM) for 30 min in extracellular medium as previous reports [39]. The aortic segments were then washed, cut, and the endothelium was placed upside down between two coverslips [40]. The green fluorescence was excited at 488 nm and imaged through a 525 nm long-path filter [41]. Furthermore, the contents of NO in aortae and primary ECs were also examined using the Griess reagent following the manufacturer’s instructions [42].

### Immunoblot and immunoprecipitation

The samples were extracted in cell lysis buffer (P0013, Beyotime, Shanghai, China) containing Tris (20 mM, pH7.5), 150 mM NaCl, 1% Triton X-100, and sodium pyrophosphate, β-glycerophosphate, EDTA, Na_3_VO_4_, and leupeptin, and then centrifuged at 12,000 g for 15 min at 4 °C, and the supernatants in each sample were collected. Total protein in each sample was quantified using a BCA protein assay kit (P0009, Beyotime Biotechnology, Shanghai, China). The protein level was normalized and the 5× loading buffer was added, following boiled for 5 min at 100 °C. Then, the equal amount of protein was electrophoresed on SDS-PAGE and transferred to PVDF membranes. After blocking for 1 h by 5% skimmed milk, the membranes were incubated with the required primary antibodies overnight at 4 °C. The membranes were then probed with horseradish peroxidase- (HRP-) conjugated secondary antibodies for 1 h, and the blot bands were visualized using enhanced chemiluminescence (WBKLS0100, Millipore, Billerica, MA, USA). The band intensities were analyzed and normalized by β–actin using ImageJ gel analysis software. For immunoprecipitation assays, the samples were lysed with 0.5 mL of cell lysis buffer, and then centrifuged for 15 min to obtain the supernatants. The supernatants were immunoprecipitated by indicated antibodies for 2 h at 4 °C, and the Protein G PLUS-Agarose was then added on a rocker platform. The precipitates were washed 3 times with lysate, and the pellets were then resuspended in electrophoresis sample buffer (40 µl) and boiled for 5 min, and the immune complexes were subjected to immunoblotting.

### CHX chase assay

Primary ECs were transfected with empty vectors or NCEH1 overexpression plasmids (sc-435,704-LAC, Santa Cruz, CA, USA) for 4 h in FBS-free DMEM, then changed to EC growth medium for additional 48 h. After that, the cells were incubated for CHX (100 µg/mL) for 0, 3, 6, 9, 12, 15, 18 24 h, respectively. The cells were collected for further immunoblotting assay.

### Real-time fluorescence quantitative polymerase chain reaction (RT-PCR)

Total RNA in each sample was extracted using the TRIzol reagent (Invitrogen, CA, USA) according to the manufacturer’s instructions, followed by the cDNA synthesis with the aid of Hifair® III 1st Strand cDNA Synthesis SuperMix (Yeasen, China). In compliance with manufacturer’s protocols, the RT-PCR was carried out using Hieff® qPCR SYBR Green Master Mix (Yeasen, China). The expression levels of the target gene were relative to β-actin and their expression was relatively quantified by the 2^−ΔΔCt^ method. The prime sequences for NCEH1: 5′-AAGGTCTTCTCCGAAAGTGAAGG-3′ (Forward), 5′-CCTCCGTGGATATAGATGACGC-3′ (Reverse); Cav-1: 5′-GCGACCCCAAGCATCTCAA-3′ (Forward), 5′-ATGCCGTCGAAACTGTGTGT-3′ (Reverse); β-actin: 5′-CCGTGAAAAGATGACCCAGA-3′ (Forward), 5′-TACGACCAGAGGCATACAG-3′ (Reverse). All primers were provided by Sangon Biotech (Shanghai, China).

### NCEH1 activity

The NCEH1 activity was determined as previously depicted [43]. In short, the mouse aortae or primary ECs were homogenized in Tris-HCl (10 mM, pH 7.0) supplemented with sucrose (250 mM) and EDTA (0.1 mM), and the mixture was centrifuged for 30 min using 43,000×g at 4 °C. The supernatants were taken as enzyme solution. Total protein in each sample was quantified using a BCA protein assay kit, and the protein in each sample was adjusted to 1 mg/ml for NCEH1 activity assay [44]. The upper layer was mixed with Scintisol and the radioactivity was counted by a liquid scintillation counter.

### Cell culture

Primary mouse aortic ECs were prepared as previously described [45–48]. In brief, the mice were anesthetized by intraperitoneal injection of pentobarbital sodium (40 mg/kg). Heparin (100U/mL in PBS) was injected from the left ventricle into the circulation to flush out the blood in the blood vessels, and the aortae was placed in Dulbecco’s modified Eagle medium (DMEM) supplemented with collagenase type II (0.8 mg/ml, Sigma) for 8 min at 37 °C. Detached ECs were obtained by centrifugation 5 min at 1000 rpm, and then re-suspended in 20% FBS-DMEM. After that, the ECs were then cultured in endothelial cell growth medium containing bovine brain extract (Lonza, Walkersville, MD, USA) until confluency. Primary ECs were transfected with adenovirus-encoding NCEH1 shRNA (100 nM, sc-140,728-V, Santa Cruz, CA, USA) or NCEH1 overexpression plasmids (1 µg, sc-435,704-LAC, Santa Cruz, CA, USA) for 6 h in FBS-free DMEM, then changed to EC growth medium in the presence or absence of HG (25 mM) for additional 48 h. Eventually, the cells were collected for further experiments. HEK293T cells were cultured in DMEM supplemented with 2 mM glutamine, 10% fetal bovine serum (Gibco), 10,000 Units/ml penicillin, and 10 mg/ml streptomycin (Gibco) in 5% CO_2_ at 37 °C. HEK293T cells were plated onto 35-mm gelatin-coated dishes and transfected with required plasmid DNA (1 µg) for 6 h using Lipofectamine 3000 (Invitrogen) according to the manufacturer’s instructions.

### Plasmid construction and cell transfection

Sequences encoding full-length NCEH1 and ZNRF1 were cloned into pcDNA3.1-Flag and pcDNA3.1-hemagglutinin (HA) vectors to yield pcDNA3.1-Flag-NCEH1 and pcDNA3.1-HA-NKAα1, respectively. Plasmids encoding pcDNA3.1-HA-NCEH1 and pcDNA3.1-FLAG-ZNRF1 were acquired by cloning the indicated cDNA of NCEH1 and ZNRF1 (non-transmembrane region fragment) into pcDNA3.1-HA and pcDNA3.1-Flag, respectively. Plasmids encoding pcDNA5-HA-GST-NCEH1 and pcDNA5-HA-GST-ZNRF1 were obtained by cloning the indicated cDNA of NCEH1and ZNRF1, respectively, into pcDNA5-HA-GST vectors. These plasmids (1 µg) were transfected to HEK293 cells for 6 h, and the complete medium was changed and cultured for additional 48 h, and their interactions were examined by immunoblotting.

### Statistical analysis

In this study, the cellular and molecular experiments were independently repeated for at least 3 times. The animal assays involved were independently repeated for at least 6 mice. Data from replications were averaged and expressed as mean value ± standard deviation (SD). All results were subjected to normal distribution test using Skewness and Kurtosis methods, and all data passed the normal distribution. The statistical analysis was conducted by GraphPad Prism 5.0 (GraphPad Software, Inc., San Diego, CA, USA). Unpaired t-test was utilized to determine differences between two groups. Analysis of variance (ANOVA) was performed for the comparison of multiple groups. Bonferroni post-hoc testing was used following ANOVA for analyzing all comparisons among groups. *P* < 0.05 was deemed as statistically significant.

## Results

### NCEH1 is dysregulated in HFD-induced mouse aortae, or high glucose (HG)-induced ex vivo aortae and primary ECs

**Fig. 1:**
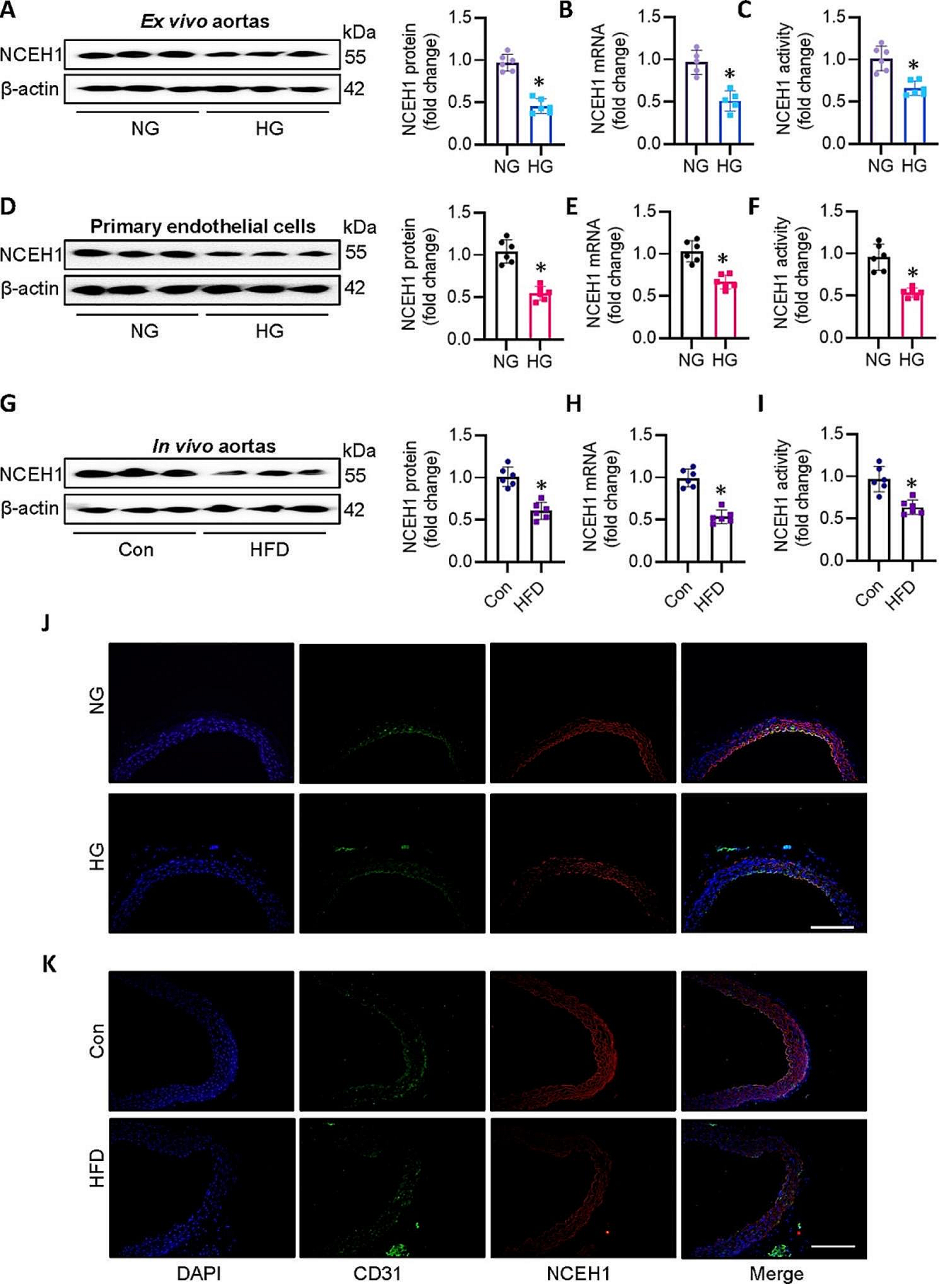
Expression of NCEH1 in HG-incubated or HFD-induced mouse aortae and HG-exposed ECs. (**A**) Representative blots and quantitation of NCEH1 protein in NG (mannitol, 25 mM) or HG (D-glucose (25 mM)-incubated mouse aortae. (**B**) Relative mRNA level of NCEH1 in NG or HG-incubated mouse aortae. (**C**) NCEH1 activity in NG or HG-incubated mouse aortae. (**D**) Representative blots and quantitation of NCEH1 protein in NG or HG-incubated ECs. (**E**) Relative mRNA level of NCEH1 in NG or HG-incubated ECs. (**F**) NCEH1 activity in NG or HG-incubated ECs. (**G**) Representative blots and quantitation of NCEH1 protein in normal diet or HFD-incubated mouse aortae. (**H**) Relative mRNA level of NCEH1 in normal diet or HFD-incubated mouse aortae. (**I**) NCEH1 activity in normal diet or HFD-incubated mouse aortae. (**J**) Immunofluorescence double staining showing the expression of NCEH1 in NG or HG-incubated mouse aortae. Scale bar, 200 µm. (**K**) Immunofluorescence double staining showing the expression of NCEH1 in normal diet or HFD-incubated mouse aortae. Scale bar, 200 µm. n = 4–6. *P < 0.05 versus Control (Con) or normal glucose (NG). The *P*-value was calculated by unpaired two-tailed Student’s t-test **(A-I)**. For immunoblotting assay, the ratio of the grayscale values of the target protein and β-actin in each group was normalized by the average value of the control group. For RT-PCR assay, the expression levels of the target gene were relative to β-actin and their expression was relatively quantified by the 2^−ΔΔCt^ method. The ratio of the activity of NCEH1 in each group was normalized to the average value of the control group. NCEH1, neutral cholesterol ester hydrolase 1; NG, normal glucose; HG, high glucose; Con, Control; HFD, high fat diet; DAPI, 4’,6-Diamidine-2’-phenylindole dihydrochloride

To investigate the potential role of NCEH1 in diabetes-induced endothelial dysfunction, we examined the activity of NCEH1 and the expression levels of NCEH1 in HG-incubated mouse aortae ex vivo, HG-induced endothelial cells in vitro, or the aortae from HFD-induced obese mice in vivo. Results showed that HG stimulation significantly reduced expression of NCEH1 at both protein and mRNA levels in mouse aortae (Fig. 1A-B), along with a decrease in NCEH1 activity (Fig. 1C). Upon exposure of HG, the protein and mRNA levels of NCEH1 were obviously decreased in primary mouse ECs (Fig. 1D-E), in conjunction with a decreased NCEH1 activity (Fig. 1F). In line with this, the decreased NCEH1 expression or activity was also observed in aortae from mice fed with a HFD when compared with those of their lean counterparts (Fig. 1G-I). Immunofluorescence double staining showed that the expression of NCEH1 in endothelial cells significantly decreased in both HG-incubated aortas (Fig. 1J) and HFD-induced mouse aortae (Fig. 1K). Overall, our studies indicated that NCEH1 expression or activity were dysfunctional in the aortas of dietary obese mice and HG-induced mouse aortae, as well as primary ECs challenged by HG.

### Role of NCEH1 in diabetes-induced endothelial dysfunction in mouse aortae

**Fig. 2:**
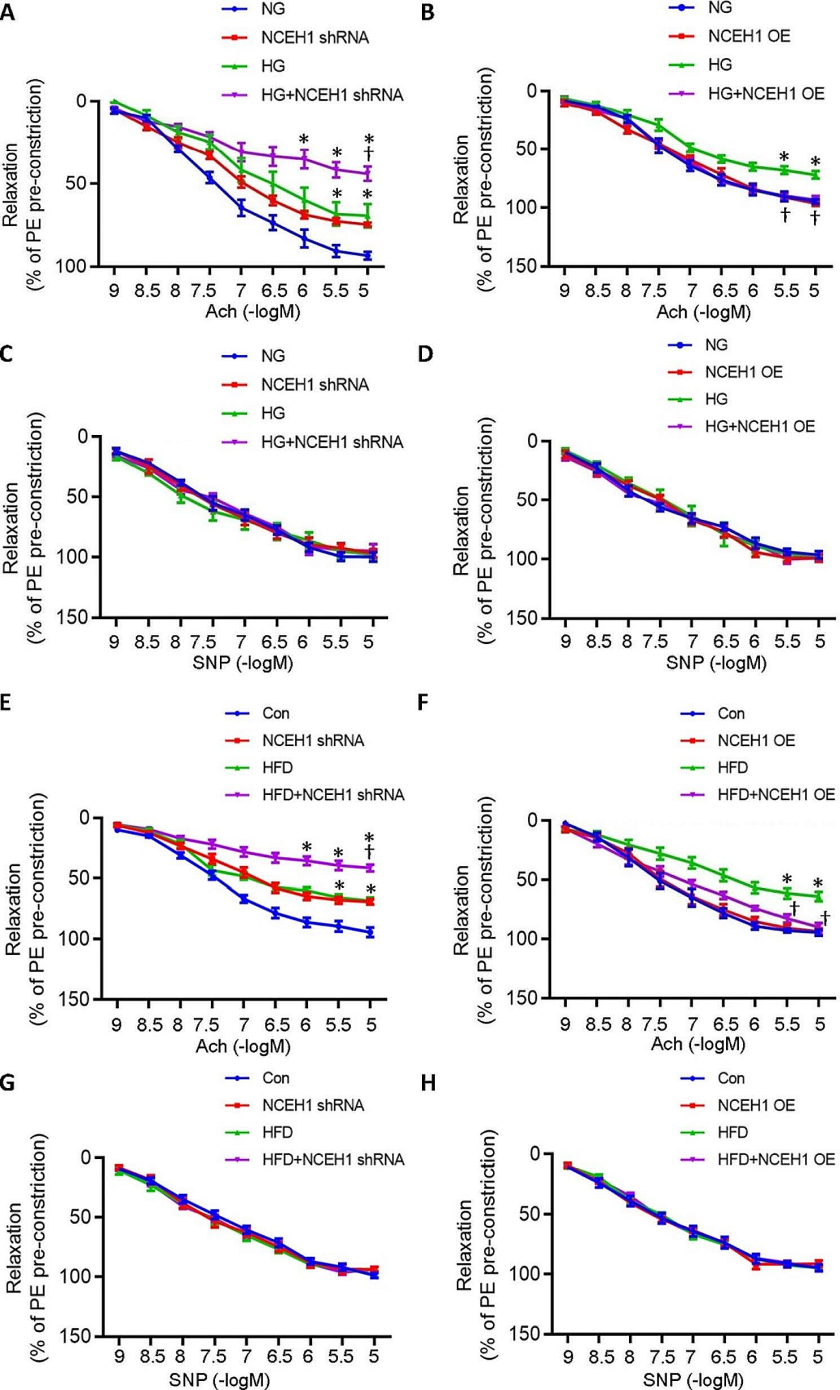
Effects of NCEH1 on vascular relaxation in HG-incubated or HFD-induced mouse aortae. (**A**) EDR in aortic rings from NG (mannitol, 25 mM) or HG (D-glucose (25 mM)-induced mouse aortae with or without NCEH1. (**B**) EDR in aortic rings from NG- or HG-induced mouse aortae after overexpression of NCEH1. (**C**) Endothelium-independent relaxation in aortic rings from NG- or HG-induced mouse aortae with or without NCEH1. (**D**) Endothelium-independent relaxation in aortic rings from NG- or HG-induced mouse aortae after overexpression of NCEH1. (**E**) EDR in aortic rings from normal diet- or HFD-induced mouse aortae with or without NCEH1. (**F**) EDR in aortic rings from normal diet- or HFD-induced mouse aortae after overexpression of NCEH1. (**G**) Endothelium-independent relaxation in aortic rings from normal diet- or HFD-induced mouse aortae with or without NCEH1. (**H**) Endothelium-independent relaxation in aortic rings from normal diet- or HFD-induced mouse aortae after overexpression of NCEH1. *n* = 6. *P < 0.05 versus Control (Con) or normal glucose (NG). †*P* < 0.05 versus HG or HFD. Differences between groups were assessed with ANOVA followed by Bonferroni post-hoc test **(A-H)**. Relaxation at each concentration was expressed as the percentage of force in response to Phe. NCEH1, neutral cholesterol ester hydrolase 1; NG, normal glucose; HG, high glucose; Con, Control; HFD, high fat diet; Phe, phenylephrine; Ach, acetylcholine; SNP, sodium nitroprusside

Endothelium-dependent relaxation (EDR) in aortic rings from NCEH1-deficient mice was inhibited when compared with control mice (Fig. 2A, S1A). HG exposure further worsened the dysfunction of EDR in NCEH1-deficient mice as compared with that from control mice (Fig. 2A). In sharp contrast, endothelial-specific overexpression of NCEH1 by adeno-associated virus (AAV) infection improved EDR in aortic rings from mice under HG conditions although EDR was similar between control and NCEH1 overexpression mice (Fig. 2B, S1B). Interestingly, addition of sodium nitroprusside (SNP), an exogenous NO donor, led to comparable and full vascular relaxations in different groups (Fig. 2C-D). Next, we further examined the role of NCEH1 in endothelial function of aortae of diet-induced obese mice. In keeping with the ex vivo results, EDR in aortae from HFD mice was reduced, this was further declined in HFD mice by specific knockdown of endothelial NCEH1 by AAV-Tie1-NCEH1. (Fig. 2E). On the contrary, tail vein injection of AAV-mediated endothelial-specific NCEH1 overexpression augmented EDR in aortae from HFD-induced mice (Fig. 2F, S1C). SNP-induced relaxation responses were not changed by endothelial-specific knockdown or overexpression of NCEH1 (Fig. 2G-H). Mice fed with HFD developed type 2 diabetes characterized by increased body weight, fasting blood glucose, fasting serum insulin, total cholesterol and triglyceride in comparison with mice fed with chow diet (Table S1-2). These changes remain unaltered in HFD mice in the presence or absence of NCEH1 (Table S1-2). These results indicated that vascular dysfunction in diabetes could be ameliorated by NCEH1 in an endothelium-dependent mechanism, which was not associated with metabolic disturbances in diabetic mice.

### NCEH1 improves EDR in HFD mice via disrupting the caveolin-1 (Cav-1)/eNOS complex

NO is a vasodilatation factor secreted by vascular endothelial cells, and reduced NO bioavailability underlies the pathogenesis of diabetes-induced endothelial dysfunction [49]. Thereby, we determined whether NCEH1 regulated EDR in a NO-dependent manner by assessing the contents of NO in mouse aortae or primary endothelial cells. The levels of NO were downregulated in mouse aortae after endothelial deletion of NCEH1 under NG conditions (Fig. S2A-B). This decrease was further exaggerated under HG environment (Fig. S2C). Conversely, endothelial-specific overexpression of NCEH1 not only enhanced NO contents at basal conditions (Fig. S2D-E), but also reversed HG-inhibited NO production in mouse aortae (Fig. S2F). The endothelial-dependent vasodilator effect involves phosphorylation of eNOS on residue Ser1177, resulting in enhanced NO production [13, 50]. To evaluate whether the eNOS phosphorylation was the mechanism by which NCEH1-induced vasodilation, we measured eNOS phosphorylation in mouse arteries. Intriguingly, the eNOS phosphorylation levels were not changed by ablation or overexpression of NCEH1 (Fig. S2G-H). Similar results were observed in primary ECs (Fig. S2I-N). These findings suggested that the amelioration of EDR by NCEH1 was not related with eNOS phosphorylation.

**Fig. 3:**
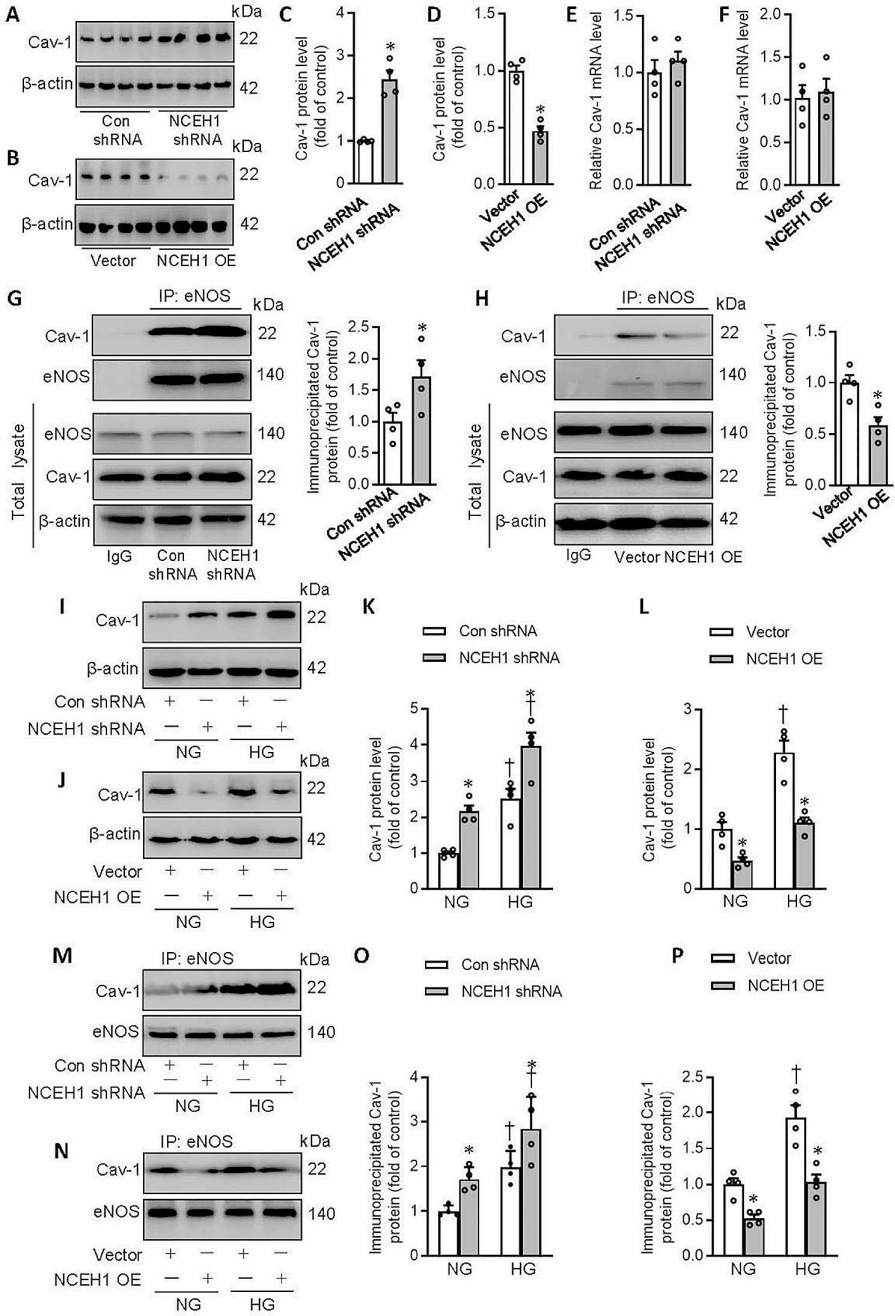
Effects of NCEH1 on the formation of Cav-1/eNOS in mouse aortae. (**A, C**) Effects of NCEH1 shRNA on the protein expression of Cav-1. (**B, D**) Effects of NCEH1 overexpression on the protein expression of Cav-1. (**E**) Effects of NCEH1 shRNA on the mRNA level of Cav-1. (**F**) Effects of NCEH1 overexpression on the mRNA level of Cav-1. (**G**) Effects of NCEH1 shRNA on the Cav-1/eNOS complex. (**H**) Effects of NCEH1 overexpression on the Cav-1/eNOS complex. (**I, K**) Effects of NCEH1 shRNA on the protein expression of Cav-1 upon HG exposure. (**J, L**) Effects of NCEH1 overexpression on the protein expression of Cav-1 upon HG exposure. (**M, O**) Effects of NCEH1 shRNA on the Cav-1/eNOS complex upon HG exposure. (**N, P**) Effects of NCEH1 overexpression on the Cav-1/eNOS complex upon HG exposure. *n* = 4. *P < 0.05 versus Con shRNA or Vector. †*P* < 0.05 versus NG. The *P*-value was calculated by unpaired two-tailed Student’s t-test **(A-H)**. Differences between groups were assessed with ANOVA followed by Bonferroni post-hoc test **(K, L,O, P)**. For immunoblotting assay, the ratio of the grayscale values of the target protein and β-actin in each group was normalized by the average value of the control group. For RT-PCR assay, the expression levels of the target gene were relative to β-actin and their expression was relatively quantified by the 2^−ΔΔCt^ method. Cav-1, caveolin-1; NCEH1, neutral cholesterol ester hydrolase 1; NG, normal glucose; HG, high glucose; OE, overexpression; eNOS, endothelial nitric oxide synthase

In the endothelium, cav-1 anchors eNOS to plasma membrane caveolae, thus limiting its cytosolic translocation and activation [51]. The formation of cav-1/eNOS complex inhibits eNOS activity which is responsible for NO production [52]. Upregulation of cav-1 contributes to high-salt diet-induced endothelial dysfunction and hypertension through decreased eNOS activation [53]. We then examined the interaction of cav-1 with eNOS in mouse aortae or primary endothelial cells. When NCEH1 is specifically reduced in the endothelium, the protein expression of cav-1 was elevated in mouse aortae (Fig. 3A, C). Endothelial-specific overexpression of NCEH1 blunted the protein expression of cav-1 (Fig. 3B, D). The transcriptional level of cav-1 was not altered by either NCEH1 knockdown or NCEH1 overexpression (Fig. 3E-F). Consistently, NCEH1 deficiency increased, while NCEH1 overexpression downregulated the formation of Cav-1/eNOS complex (Fig. 3G-H). Under HG circumstances, the protein expression of cav-1 (Fig. 3I, K) and cav-1/eNOS complex (Fig. 3M, O) was further augmented in mouse aortae subjected to silencing NCEH1. Nevertheless, overexpression of NCEH1 exhibited the opposite effects on the protein expression of cav-1 (Fig. 3J, L) and cav-1/eNOS complex (Fig. 3N, P). The results were replicated in control and HFD mice (Fig. S3).

**Fig. 4:**
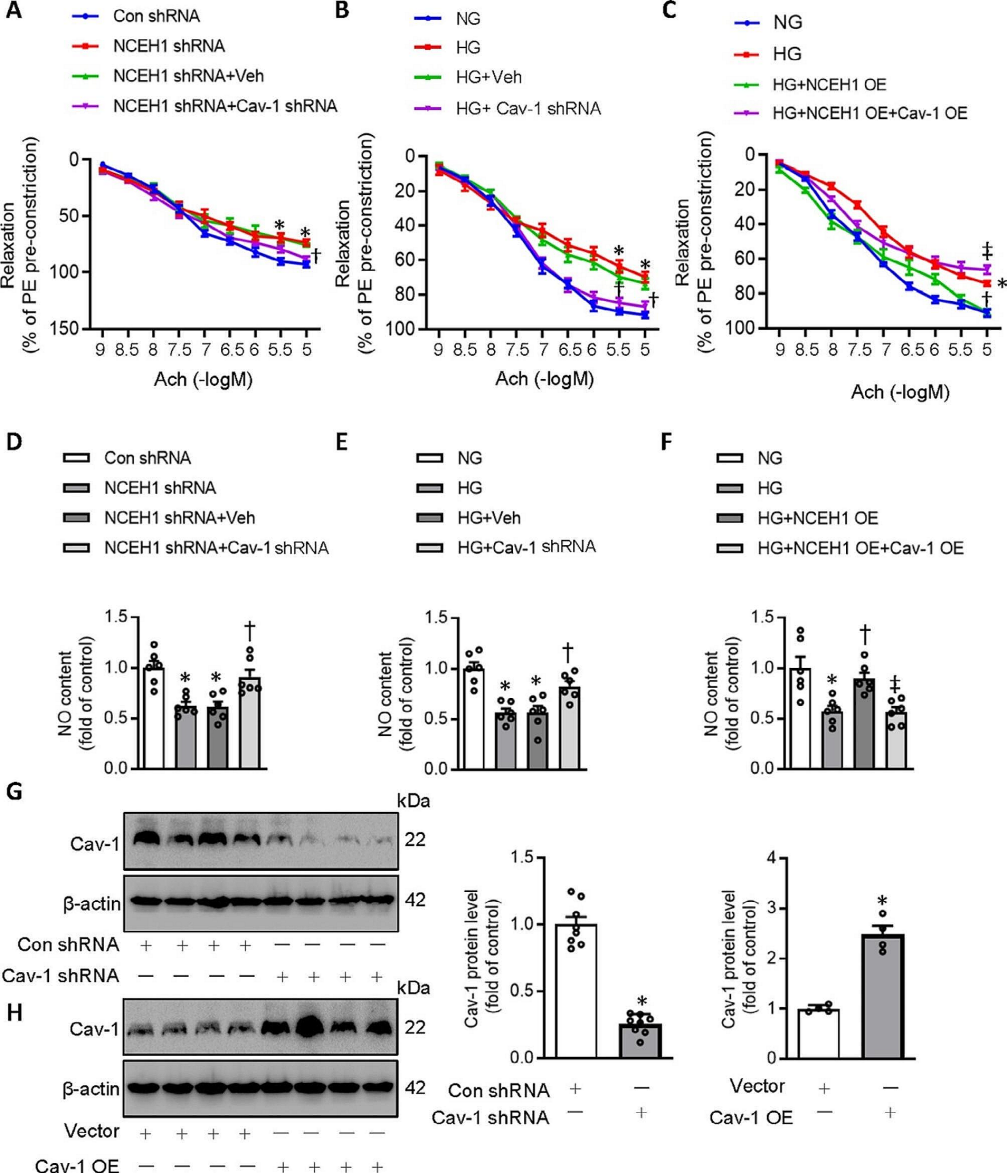
Overexpression of Cav-1 abolished the vascular benefits of NCEH1 overexpression in mouse aortae. (**A**) Silencing Cav-1 improved EDR in NCEH-1 deficient mouse aortae. (**B**) Silencing Cav-1 improved EDR in HG-exposed mouse aortae. (**C**) Overexpression of Cav-1 attenuated the effects of NCEH1 overexpression on EDR in HG-exposed mouse aortae. (**D**) Silencing Cav-1 restored NO contents in NCEH-1 deficient mouse aortae. (**E**) Silencing Cav-1 restored NO contents in HG-exposed mouse aortae. (**F**) Overexpression of Cav-1 attenuated the effects of NCEH1 overexpression on NO production in HG-exposed mouse aortae. (**G**) Efficiency detection of Cav-1 knockdown. (**H**) Efficiency detection of Cav-1 overexpression. *n* = 4–6. *P < 0.05 versus Con shRNA or Vector. †*P* < 0.05 versus NEH1 shRNA or HG. ‡ *P* < 0.05 versus HG + NCEH1 overexpression (OE). Differences between groups were assessed with ANOVA followed by Bonferroni post-hoc test **(A-F)**. The *P*-value was calculated by unpaired two-tailed Student’s t-test **(G-H)**. For immunoblotting assay, the ratio of the grayscale values of the target protein and β-actin in each group was normalized by the average value of the control group. Relaxation at each concentration was expressed as the percentage of force in response to Phe. NCEH1, neutral cholesterol ester hydrolase 1; NG, normal glucose; HG, high glucose; OE, overexpression; NO, nitric oxide; Phe, phenylephrine; Ach, acetylcholine

Isometric study showed the impairment of acetylcholine (Ach)-induced relaxations in aortas of both NCEH1-deficient aortae and HG-incubated aortae (Fig. 4A-B, G). Knockdown of cav-1 markedly improved Ach-induced relaxations in both NCEH1-deficient aortae and HG-incubated aortae (Fig. 4A-B, G). Importantly, the improvement of NCEH1 overexpression on EDR was largely attenuated by overexpression of cav-1 in HG-incubated mouse aortae (Fig. 4C). Knockdown of Cav-1 restored the contents of NO in both NCEH1-deficient aortae and HG-incubated aortae (Fig. 4D-E). Overexpression of cav-1 abolished the effects of NCEH1 overexpression on NO contents (Fig. 4F, H). These results were reproduced in primary ECs (Fig. S4). These results hinted that NCEH1 protects endothelial function in diet-induced obese mice by dissociating eNOS from cav-1, allowing eNOS activation and NO generation.

### NCEH1 promotes the ubiquitination and degradation of cav-1

**Fig. 5:**
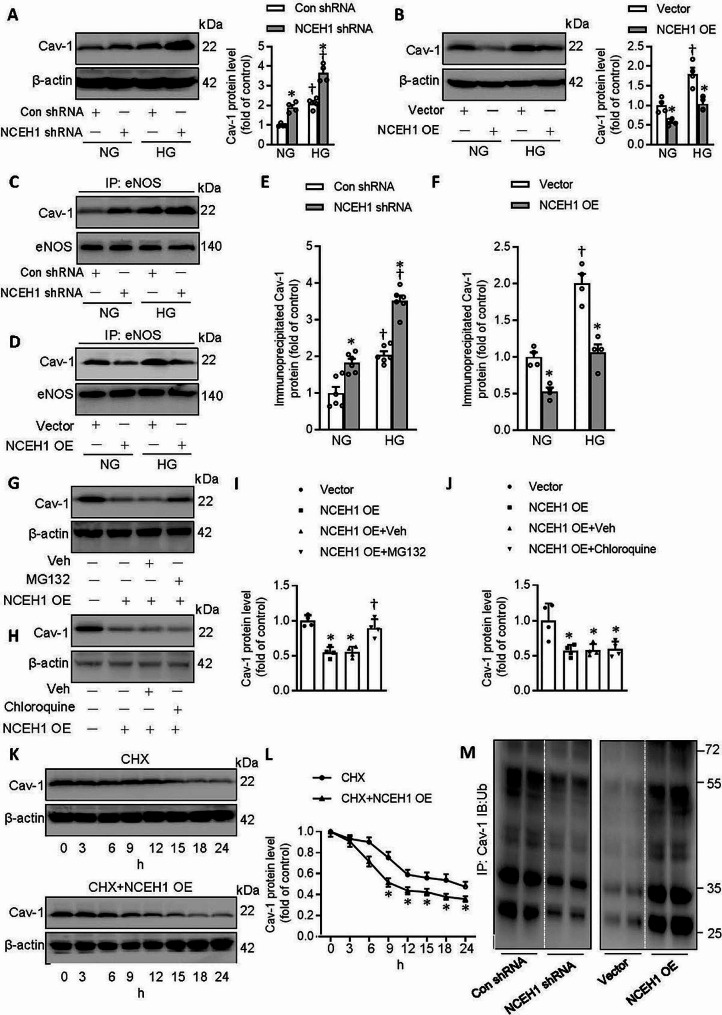
NCEH1 upregulated the expression of Cav-1 through the deubiquitination pathway in ECs. (**A**) Effects of NCEH1 shRNA on the protein expression of Cav-1. (**B**) Effects of NCEH1 overexpression on the protein expression of Cav-1. (**C, E**) Effects of NCEH1 shRNA on the Cav-1/eNOS complex. (**D, F**) Effects of NCEH1 overexpression on the Cav-1/eNOS complex. (**G, I**) MG-132 reversed the inhibitory effects of NCEH1 overexpression on the protein expression of Cav-1. (**H, J**) Chloroquine had no effect on the inhibitory effects of NCEH1 overexpression on the protein expression of Cav-1. (**K, L**) NCEH1 overexpression accelerated the degradation of Cav-1. (**M**) Effects of NCEH1 downregulation or overexpression on the ubiquitination levels of Cav-1 after transfection of NCEH1 shRNA or NCEH1 overexpression plasmid for 48 h. *n* = 4–6. *P < 0.05 versus Con shRNA, Vector or CHX. †*P* < 0.05 versus NCEH1 shRNA, NG or NCEH1 OE. Differences between groups were assessed with ANOVA followed by Bonferroni post-hoc test **(A-J)**. The *P*-value was calculated by unpaired two-tailed Student’s t-test **(L)**. For immunoblotting assay, the ratio of the grayscale values of the target protein and β-actin in each group was normalized by the average value of the control group. NCEH1, neutral cholesterol ester hydrolase 1; NG, normal glucose; HG, high glucose; OE, overexpression; NO, nitric oxide; Cav-1, caveolin-1; eNOS, endothelial nitric oxide synthase; CHX, cycloheximide; IP, co-immunoprecipitation

In line with ex vivo results, the protein expression of cav-1 was obviously elevated in ECs exposed to HG, effects that were aggravated by deficiency of NCEH1 (Fig. 5A), but were reversed by overexpression of NCEH1 (Fig. 5B). Correspondingly, NCEH1-deficient ECs exhibited higher levels of the Cav-1/eNOS complex in both NG and HG conditions (Fig. 5C, E). Compared with ECs exposed to HG, the formation of the Cav-1/eNOS complex was considerably attenuated by NCEH1 overexpression (Fig. 5D, F).

As is known, there are two signaling pathways are involved in the degradation of intercellular proteins, such the ubiquitin-proteasome system and the lysosome system [54]. To investigate which pathway participated in NCEH1-induced Cav-1 degradation, we treated with ECs with lysosome and proteasome inhibitors. As shown in Fig. 5G and I, pretreatment with proteasome inhibitors MG-132 (10 µM) dramatically reversed NCEH1-induced Cav-1 degradation. However, the lysosome inhibitor chloroquine (100 µM) did not reverse the degradation of Cav-1 induced by NCEH1 overexpression (Fig. 5H and J). Moreover, we conducted CHX (100 µg/mL) chase assays to evaluate the half-life of Cav-1 in ECs. We found that the half-life of Cav-1 was much shorter in ECs after NCEH1 overexpression, suggesting that NCEH1 stimulated the degradation of Cav-1 through accelerating the half-life of Cav-1 in ECs (Fig. 5K and L).

A large amount of ubiquitinated Cav-1 was present in normal ECs, while the polyubiquitinated Cav-1 was significantly removed by silencing NCEH1 (Fig. 5M). In sharp contrast, NCEH1 overexpression enhanced the ubiquitin conjugation of Cav-1 (Fig. 5M). NCEH1 downregulation further potentiated, while NCEH1 overexpression prevented HG-induced Cav-1 deubiquitination in primary ECs (Fig. S5). These results indicated that NCEH1 promoted the ubiquitination and degradation of Cav-1 in ECs.

### Zinc and ring finger 1 (ZNRF1) is required for NCEH1 to promote the ubiquitination and degradation of Cav-1

**Fig. 6:**
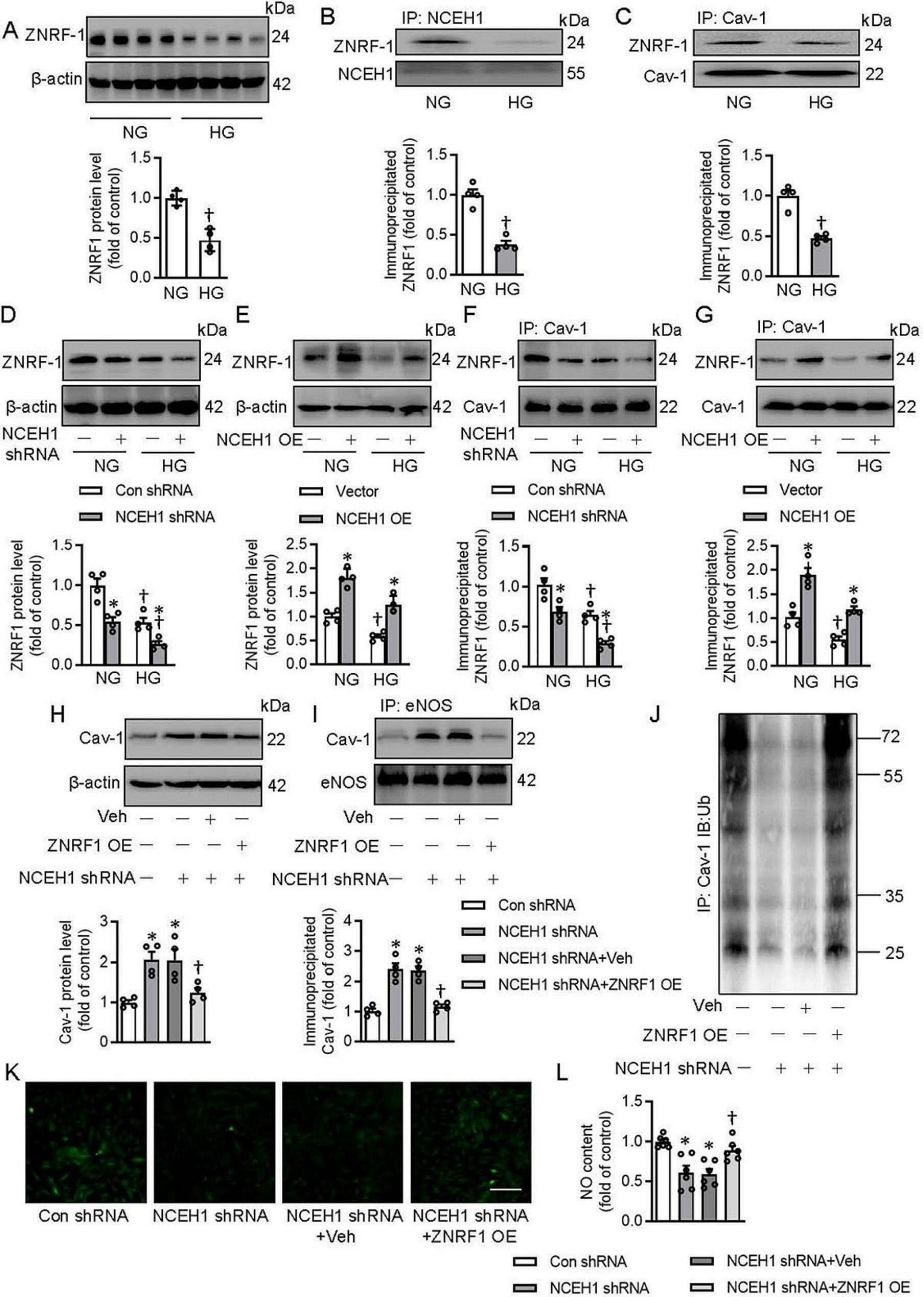
NCEH1 required ZNRF1 to induce the ubiquitination-dependent degradation of Cav-1 in ECs. (**A**) The protein expression of ZNRF1 in NG- or HG-exposed ECs. (**B**) The interaction of ZNRF1 with NCEH1 in NG- or HG-exposed ECs. (**C**) The interaction of ZNRF1 with Cav-1 in NG- or HG-exposed ECs. (**D**) Effects of NCEH1 shRNA on the protein expression of ZNRF1 in NG- or HG-exposed ECs. (**E**) Effects of NCEH1 overexpression on the protein expression of ZNRF1 in NG- or HG-exposed ECs. (**F**) Effects of NCEH1 shRNA on the interaction of ZNRF1 with Cav-1 in NG- or HG-exposed ECs. (**G**) Effects of NCEH1 overexpression on the interaction of ZNRF1 with Cav-1 in NG- or HG-exposed ECs. (**H**) Effects of ZNRF1 overexpression on the protein expression of Cav-1 in NCEH1-deficient ECs. (**I**) Effects of ZNRF1 overexpression on the Cav-1/eNOS complex in NCEH1-deficient ECs. (**J**) Primary ECs were transfected with ZNRF1 overexpression plasmid (1 µg) for 6 h, and then transfected with NCEH1 shRNA (100 nM) for additional 48 h, we then examined the effects of ZNRF1 overexpression on ubiquitination of Cav-1 in NCEH1-deficient ECs. (**K, L**) Effects of ZNRF1 overexpression on NO production in NCEH1-deficient ECs. Scale Bar, 100 μm. *n* = 4–6. *P < 0.05 versus Con shRNA or Vector. †*P* < 0.05 versus NCEH1 shRNA. The *P*-value was calculated by unpaired two-tailed Student’s t-test (**A-C**). Differences between groups were assessed with ANOVA followed by Bonferroni post-hoc test (**D-L**). For immunoblotting assay, the ratio of the grayscale values of the target protein and β-actin in each group was normalized by the average value of the control group. Veh, Vehicle. NCEH1, neutral cholesterol ester hydrolase 1; NG, normal glucose; HG, high glucose; OE, overexpression; NO, nitric oxide; Cav-1, caveolin-1; eNOS, endothelial nitric oxide synthase; IP, immunoprecipitation

E3 ubiquitin-protein ligase ZNRF1 is documented to promote caveolin-1 ubiquitination and degradation in immune responses [55]. Thus, we next investigated whether NCEH1 reduced Cav-1 deubiquitination via regulating ZNRF1. The protein interaction database Genemania demonstrated the direct interaction of ZNRF1 with NCEH1 (http://genemania.org/search/, Fig. S6A). The interaction of ZNRF1 with NCEH1 was also confirmed by the hitpredict database (Table S3). The co-IP assays further demonstrated that NCEH1 interacted with ZNRF1 directly (Fig. S6B-E). The protein expression of ZNRF1 was lower in HG-exposed ECs (Fig. 6A). The interaction of NCEH1 with ZNRF1 or the ZNRF1/Cav-1 complex was downregulated in ECs after HG treatment (Fig. 6B-C). Deficiency of NCEH1 further decreased, whereas overexpression of NCEH1 restored the protein expression of ZNRF1 in HG-challenged ECs (Fig. 6D-E). Likewise, the formation of ZNRF1/Cav-1 complex was further diminished in NCEH1-deficient ECs in the context of HG (Fig. 6F). In contrast, overexpression of NCEH1 enhanced the interaction of ZNRF1 with Cav-1 in ECs under both NG and HG conditions (Fig. 6G). Importantly, the protein expression of Cav-1 and the Cav-1/eNOS complex were attenuated by overexpression of ZNRF1 in ECs when NCEH1 was absent (Fig. 6H-I). The deubiquitination of Cav-1 disappeared when ECs were transfected with ZNRF1 plasmids (Fig. 6J). Ectopic overexpression of NCEH1 conserved the contents of NO in NCEH1-deficient ECs (Fig. 6K-L).

ZNRF1 downregulation abolished the effects of NCEH1 overexpression on NO generation in HG-incubated ECs (Fig. S7A, S8C-D), while ZNRF1 overexpression prevented HG-induced inhibition of NO in ECs (Fig. S7B, S8A-B). Similar to what we observed in NCEH1-deficient cells, overexpression of ZNRF1 repressed the protein expression of Cav-1, the Cav-1/eNOS complex, and the deubiquitination of Cav-1 in HG-stimulated ECs (Fig. S8E-G). On the contrary, deletion of ZNRF1 blocked the effects of NCEH1 overexpression on the protein expression of Cav-1, the Cav-1/eNOS complex, and the deubiquitination of Cav-1 (Fig. S8H-J). Similar to cellular results, ZNRF1 was responsible for NCEH1 to evoke ubiquitination-dependent degradation of Cav-1, activation of eNOS, and release of NO in isolated blood vessels (Fig. S9). These results collectively indicated that NCEH1 recruited ZNRF1 which was responsible for the ubiquitination and degradation of Cav-1 in ECs, leading to the disruption of the Cav-1/eNOS complex and subsequent NO production.

### ZNRF1 knockdown attenuates the benefits of NCEH1 on endothelial dysfunction in mouse aortae

**Fig. 7:**
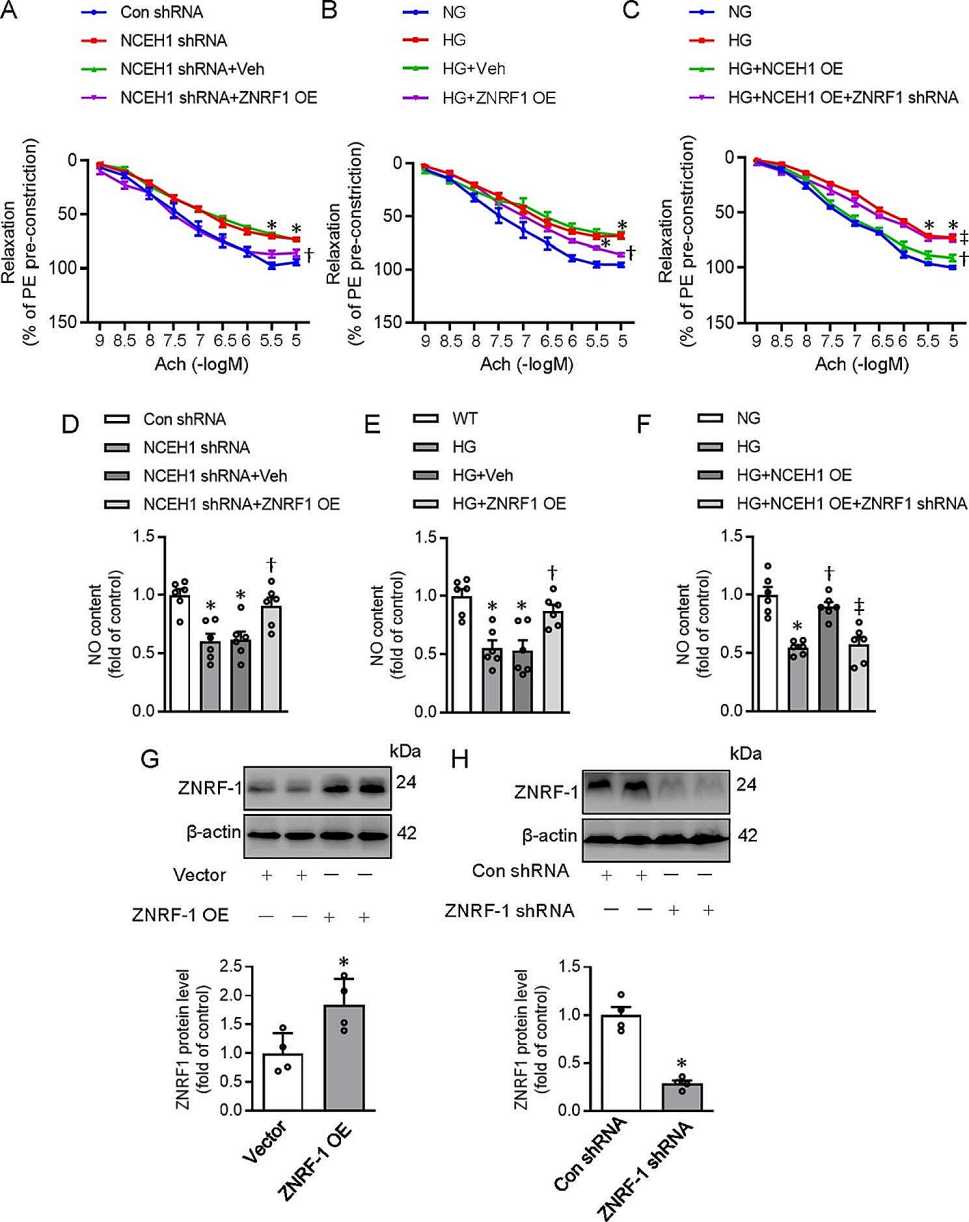
Effects of NCEH1 on the formation of Cav-1/eNOS in mouse aortae. (**A**) ZNRF1 overexpression improved EDR in NCEH-1 deficient mouse aortae. (**B**) ZNRF1 overexpression improved EDR in HG-exposed mouse aortae. (**C**) Silencing ZNRF1 attenuated the effects of NCEH1 overexpression on EDR in HG-exposed mouse aortae. (**D**) ZNRF1 overexpression restored NO contents in NCEH-1 deficient mouse aortae. (**E**) ZNRF1 overexpression restored NO contents in HG-exposed mouse aortae. (**F**) Silencing ZNRF1 attenuated the effects of NCEH1 overexpression on NO production in HG-exposed mouse aortae. (**G**) Efficiency detection of ZNRF1 overexpression. (**H**) Efficiency detection of ZNRF1 knockdown. *n* = 4–6. *P < 0.05 versus Con shRNA or Vector. †*P* < 0.05 versus NCEH1 shRNA or HG. ‡ *P* < 0.05 versus HG + NCEH1 overexpression (OE). Differences between groups were assessed with ANOVA followed by Bonferroni post-hoc test (**A-F**). The *P*-value was calculated by unpaired two-tailed Student’s t-test (**G, H**). For immunoblotting assay, the ratio of the grayscale values of the target protein and β-actin in each group was normalized by the average value of the control group. Relaxation at each concentration was expressed as the percentage of force in response to Phe. NCEH1, neutral cholesterol ester hydrolase 1; NG, normal glucose; HG, high glucose; OE, overexpression; NO, nitric oxide; Phe, phenylephrine; Ach, acetylcholine

The protein expression of ZNRF1 was downregulated in HG-incubated isolated aortae and HFD-induced aortae (Fig. S10A-B). The protein expression of ZNRF1 and the ZNRF1/Cav-1 complex were declined in HG-exposed artery rings, which were further aggravated by knockdown of NCEH1 (Fig. S10C, E). Overexpression of NCEH1 displayed the opposite effects (Fig. S10D, F). These findings were recapitulated in thoracic aorta of HFD mice (Fig. S10G-J). The concentration-dependent vasodilation in response to an endothelium-dependent vasodilator Ach was impaired NCEH1-deficient aortae or HG-exposed aortae, an effect was reversed by overexpression of ZNRF1 by adenoviral infection (Fig. 7A-B, G). Interestingly, knockdown of ZNRF1 weaken the protection of NCEH1 overexpression against endothelial dysfunction in HG-insulted mouse aortae ex vivo (Fig. 7C, H). In addition, ZNRF1 overexpression preserved the contents of NO in NCEH1-deficient aortae or HG-exposed aortae (Fig. 7D-E), while ZNRF1 downregulation abated the effects of NCEH1 overexpression on NO formation in HG-stimulated mouse aortae (Fig. 7F).

## Discussion

The current study highlighted a functional role for NCEH1 in ameliorating endothelial dysfunction in obese diabetic mice (Fig. S11). Downregulation of NCEH1 exaggerated the impaired vasodilatation induced by HG exposure or HFD, whereas NCEH1 overexpression protected against such dysfunction by disrupting the Cav-1/eNOS complex. Moreover, interaction of NCEH1 with ZNRF1, an E3 ubiquitin-protein ligase, triggered the ubiquitination and degradation of Cav-1, relieving eNOS from the inhibitory clamp of Cav-1, leading to eNOS activation and NO release in ECs. Importantly, silencing Cav-1 and upregulating ZNRF1 exhibited similar vasoprotective benefits as NCEH1 overexpression. Thus, we provided evidence showing that NCEH1 may play a therapeutic role in vascular complications associated with obesity and diabetes.

Lipid-rich plaques are reflected by a plethora of CE-laden foam cells, which are prone to rupture [56]. The hydrolysis of intracellular CE plays an important role in reversing cholesterol transport [57]. Up to now, three enzymes have been proposed to serve as neutral CE hydrolases (NCEH) in macrophages, including hormone-sensitive lipase (LIPE), cholesteryl ester hydrolase (CEH) and NCEH1 [28]. NCEH1 is ubiquitously expressed in peritoneal macrophages and atherosclerotic lesions, its overexpression prevents the deposition of CE in THP-1 macrophages and attenuates the progression of atherosclerosis in mice [28, 58]. It remains obscure whether NCEH1 has a favorable role in vasomotor function and EDR in diet-induced diabetes. Our results observed decreases in mRNA and protein expressions of NCEH1 as well as its activities in HG-incubated aortae or primary ECs, and HFD-induced obese diabetic mice. We then examined for the first time the potential effect of NCEH1 on endothelial function in obese diabetic mice by using both gain-of-function and loss-of-function strategies. Results showed that NCEH1 overexpression improved the impaired vasodilatation in mouse aortae induced by HG exposure, whereas NCEH1 downregulation exaggerated this dysfunction. After assessing the ex vivo effects, we examined the in vivo actions of NCEH1 in DIO mice, and we found that the impairment of vasodilatation was further worsened in NCEH1-deficient mice, while NCEH1 overexpression restored the vasodilatation in obese diabetic mice. These in vivo, in vitro, and ex vivo findings collectively suggest that NCEH1 deficiency is more likely to be involved in the pathogenesis of endothelial dysfunction in obese diabetic mice.

Reduced NO bioavailability has been recognized as a key priming factor for endothelial dysfunction in obesity and diabetes [59–61]. EDR is regulated by three main molecules, NO, prostacyclin and endothelium-derived hyperpolarization factors [62, 63]. NO is produced via the oxidation of L-arginine into L-citrulline by eNOS, and it induces vasorelaxation by activating the soluble guanylyl cyclase/cyclic guanosine-3,5-monophosphate pathway [64]. Mounting evidence points to malfunction in Cav-1 as a contributor to endothelial dysfunction in diabetes since increased expression of Cav-1 leads to a reduced EDR by impairing NO bioavailability [65]. Cav-1 is a resident caveolae protein that negatively regulates eNOS through its ability to interact with eNOS. An enhanced expression of Cav-1 is observed in the aortas of streptozotocin-induced diabetic rats, with concomitant decreases in eNOS activity and NO synthesis [66]. A better understanding of the role played by Cav-1 in eNOS regulation might reveal new pathogenic mechanisms linking diabetic vascular complications. In this study, we found an increased expression of Cav-1, the endogenous eNOS inhibitory protein, in HG-incubated mouse aortae or primary ECs, and HFD-induced obese diabetic mice. Deficiency of NCEH1 potentiated, while overexpression of NCEH1 attenuated HG- or HFD-induced Cav-1 expression, Cav-1/eNOS complex formation in aortas or ECs. In a separate set of experiments, silencing Cav-1 improved the impaired EDR in HG-incubated mouse aortae, whereas upregulating Cav-1 abolished the benefits of NCEH1 overexpression. The contents of NO in aortas and ECs were decreased in HG-exposed mouse aortae or primary ECs, and HFD-induced aortae, this effect correlated with an increased Cav-1 expression coupled to a reduction in eNOS activity. This phenomenon was reversed by NCEH1 overexpression, but deteriorated by NCEH1 downregulation. To this end, NCEH1 improved EDR in diabetic aortic segments by interfering with the protein-protein interaction Cav-1/eNOS, led to an increased eNOS activity, thus enhancing NO formation. Interestingly, Chen et al. found that the skeletal muscle biopsies exhibited approximately 50% less Cav-1 and eNOS expression in skeletal muscle biopsies from patients with type 2 diabetes [67]. They also showed that the semiquantitative ratio of Cav-1 to eNOS expression was ∼200 in human ECs, indicating that Cav-1 is 200-fold more abundant than eNOS in ECs [67]. Silencing Cav-1 reduced eNOS protein and gene expression in association with a two-fold increase in eNOS phosphorylation [67], indicating that downregulation of Cav-1 is required for eNOS phosphorylation and activation in human ECs. However, we found that NCEH1 had no impact on eNOS phosphorylation and activation, but changed the expression of Cav-1 and the formation of Cav-1/eNOS complex. Our results indicated that NCEH1 led to decreased NO production in ECs independently of eNOS phosphorylation and activation. However, these inconsistent results might arise the possibility that NCEH1 affected of the monomer/oligomer ratio of Cav-1 or the S-nitrosylation of Cav-1, which deserved in-depth studies. In addition, whether NCEH1 influenced the pathway of eNOS activation to produce NO, independently of the phosphorylation of threonine-495 or serine-1177 within eNOS, warrants further investigations. It will be intriguingly to know whether the eNOS: caveolin-1 ratio in these models was changed, thus demonstrating reciprocal regulatory relationship between eNOS and Cav1 in the ECs, this needs more solid evidence in the future studies.

ZNRF1 belongs to the largest class of RING-finger E3 ligases in mammals, and it has been demonstrated that ZNRF1 is involved in Wallerian degeneration via the ubiquitin-proteasome system-mediated AKT degradation [68]. It has been revealed that ZNRF1 regulates the immune response by regulating Cav-1 ubiquitination and degradation [55]. Coincidentally, we found that manual regulation of NCEH1 controlled the translational level of Cav-1, but did not govern the transcription level of Cav-1 in ECs. This drives a hypothesis that NCEH1 might regulate the expression of Cav-1 in a posttranslational modification-dependent manner. Based on the previous reports, we examined whether NCEH1 controls Cav-1 ubiquitination and degradation through modulating ZNRF1. With the aid of Genemania and Hitpredict database, we surprisingly found that NCEH1 might interact with ZNRF1 in a direct way. The co-IP and double immunofluorescence staining confirmed the interaction of NCEH1 with ZNRF1, which supported the results of bioinformatics analysis. As anticipated, ZNRF1 overexpression reversed the effects of NCEH1 deficiency or HG on the expression of Cav-1 and Cav-1/eNOS complex, as well as Cav-1 ubiquitination. These results were also seen in mouse aortae after HFD feeding. More importantly, overexpression of ZNRF1 restored ACh-induced relaxation disturbance in HG-induced mouse aortas by disrupting the Cav-1/eNOS complex and increasing NO formation in ECs. These data indicate that the endothelial protective effects of NCEH1 in obese diabetic mice were ascribed to its interaction with ZNRF1, which induced the ubiquitination-dependent degradation of Cav-1, inducing the liberation of eNOS and NO release. Notably, further studies are warranted to examine the time course effects of NCEH1 overexpression on the Cav-1 ubiquitination. This would shed light to the life span of NCEH1 mode of activity in vivo, as possible therapeutic targets for cardiovascular diseases.

In this current study, neither NCEH1 knockdown nor NCEH1 overexpression affected lipid profile and glucose metabolism in diet-induced diabetic mice. These findings suggested that the beneficial effects of NCEH1 on the endothelium are thus unlikely to be related to its metabolic regulations. The complicated role of NCEH1 in the different tissues, such as adipose tissue, liver, and skeletal muscles, warrants further studies.

## Conclusions

In conclusion, our in vivo, in vitro and ex vivo results demonstrated that NCEH1 recruited the E3 ubiquitin-protein ligase ZNRF1 to interfere with the protein-protein interaction Cav-1/eNOS, resulting in increased eNOS availability and NO production. The vascular benefits of NCEH1 make it a promising target for the treatment of endothelial dysfunction-related complications in diabetes.

### Electronic supplementary material

Below is the link to the electronic supplementary material.

Supplementary Material 1

## Data Availability

No datasets were generated or analysed during the current study.

## Abbreviations

Ach: acetylcholine
Cav-1: caveolin-1
CE: cholesteryl ester
CHX: cycloheximide
ECs: endothelial cells
EDR: endothelium-dependent relaxation
eNOS: endothelial nitric oxide synthase
HA: hemagglutinin
HFD: high-fat diet
HG: high glucose
HRP: horseradish peroxidase
LAL: lysosome acid lipase
NCEH1: neutral cholesterol ester hydrolase 1
NG: normal glucose
NO: nitric oxide
Phe: phenylephrine
PSS: physiological saline solution
RT-PCR: Real-time fluorescence quantitative polymerase chain reaction
SD: standard deviation
SNP: sodium nitroprusside
