# Supplementary material for: Deficiency of neutral cholesterol ester hydrolase 1 (NCEH1) impairs endothelial function in diet-induced diabetic mice
Source: Cardiovasc Diabetol. 2024 Apr 25;23:138. doi: 10.1186/s12933-024-02239-6 (PMC11046792; doi:10.1186/s12933-024-02239-6)
Supplement: Supplementary file 1 — Supplementary Material 1 [file 12933_2024_2239_MOESM1_ESM.docx]

**Supplementary data**

**
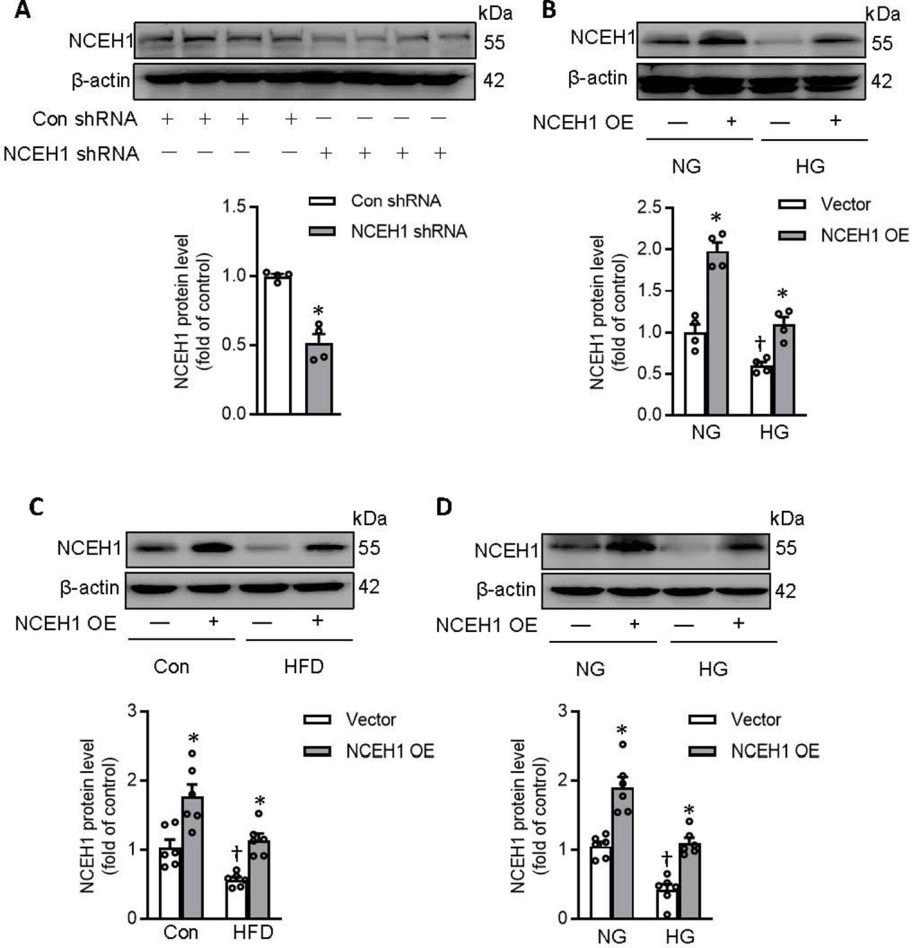
**

**Figure S1. Expression of NCHE1 in mouse aortae.** (**A**) Efficiency detection of NCEH1 knockdown in mouse aortae. (**B**) Efficiency detection of NCEH1 overexpression in HG-exposed mouse aortae. (**C**) Efficiency detection of NCEH1 overexpression in HFD-induced mouse aortae. (**D**) Efficiency detection of NCEH1 overexpression in HG-induced ECs. n=4. *P < 0.05 versus Con shRNA or Vector. †*P* < 0.05 versus NG or Con. The P-value was calculated by unpaired two-tailed Student’s t-test (A). Differences between groups were assessed with ANOVA followed by Bonferroni post-hoc test (B-D). For immunoblotting assay, the ratio of the grayscale values of the target protein and β-actin in each group was normalized by the average value of the control group. NCEH1, neutral cholesterol ester hydrolase 1; NG, normal glucose; HG, high glucose; OE, overexpression; HFD, high-fat diet.

**
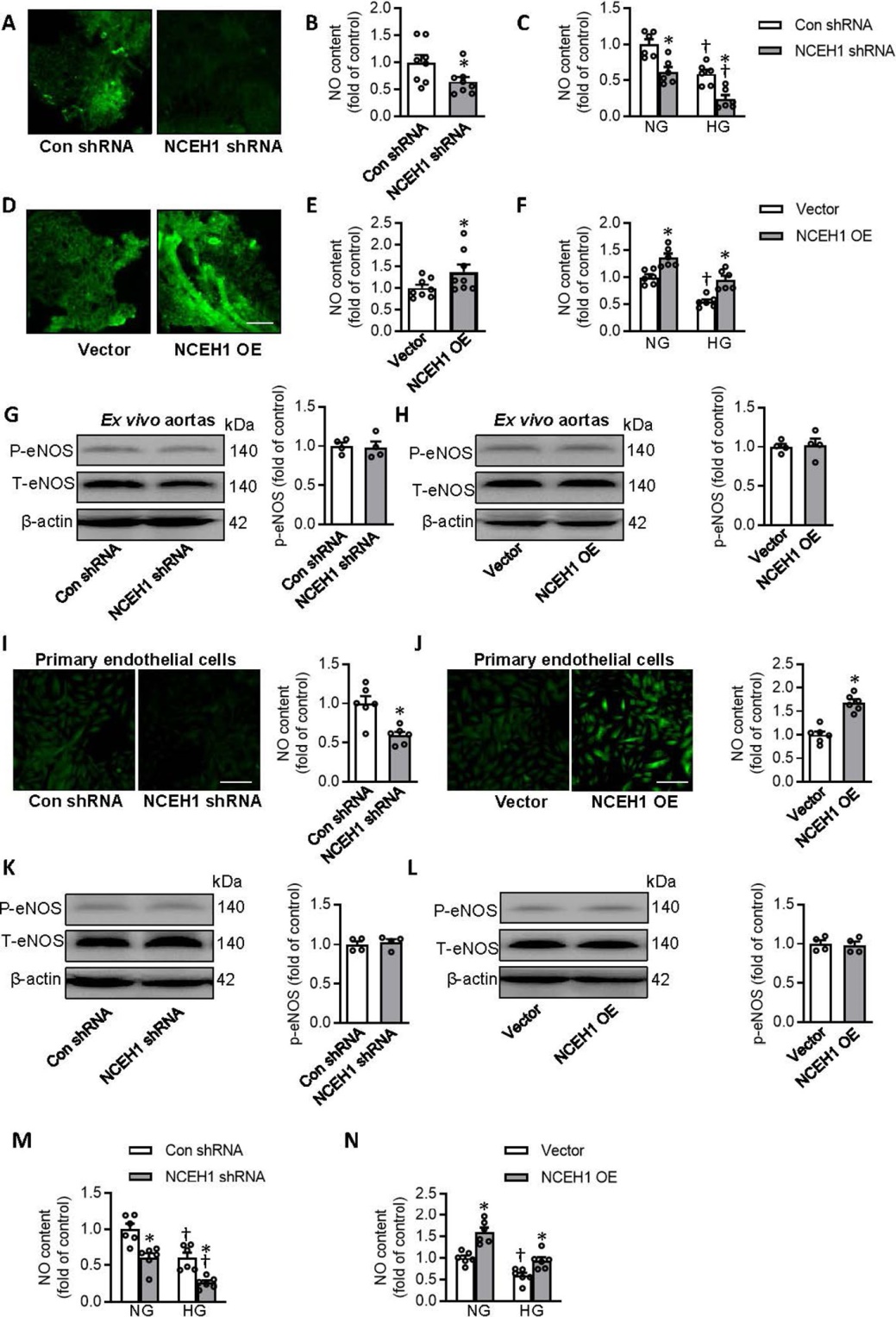
**

**Figure S2. Effects of NCEH1 on eNOS activation in *ex vivo* aortae and ECs.** (**A,B**) Effects of NCEH1 knockdown on NO production in the *en face* endothelium of aorta. Scale Bar, 100 μm. (**C**) Effects of NCEH1 knockdown on NO production in the aorta in the presence or absence of HG. (**D,E**) Effects of NCEH1 overexpression on NO production in the *en face* endothelium of aorta. (**F**) Effects of NCEH1 overexpression on NO production in the aorta in the presence or absence of HG. (**G**) Effects of NCEH1 knockdown on phosphorylated eNOS in the aorta. (**H**) Effects of NCEH1 overexpression on phosphorylated eNOS in the aorta. (**I**) Effects of NCEH1 knockdown on NO production in ECs. (**J**) Effects of NCEH1 overexpression on NO production in ECs. (**K**) Effects of NCEH1 knockdown on phosphorylated eNOS in ECs. (**L**) Effects of NCEH1 overexpression on phosphorylated eNOS in ECs. (**M**) Effects of NCEH1 knockdown on NO production in ECs in the presence or absence of HG. (**N**) Effects of NCEH1 overexpression on NO production in ECs in the presence or absence of HG. The P-value was calculated by unpaired two-tailed Student’s t-test (B,E,G-L). Differences between groups were assessed with ANOVA followed by Bonferroni post-hoc test (C,F,M,N). For immunoblotting assay, the ratio of the grayscale values of the target protein and β-actin in each group was normalized by the average value of the control group. For immunofluorescence staining, the fluorescence intensity value of each group was normalized to the fluorescence intensity average of the control group. NCEH1, neutral cholesterol ester hydrolase 1; NG, normal glucose; HG, high glucose; OE, overexpression; HFD, high-fat diet; ECs, endothelial cells; NO, nitric oxide; eNOS, endothelial nitric oxide synthase.

**
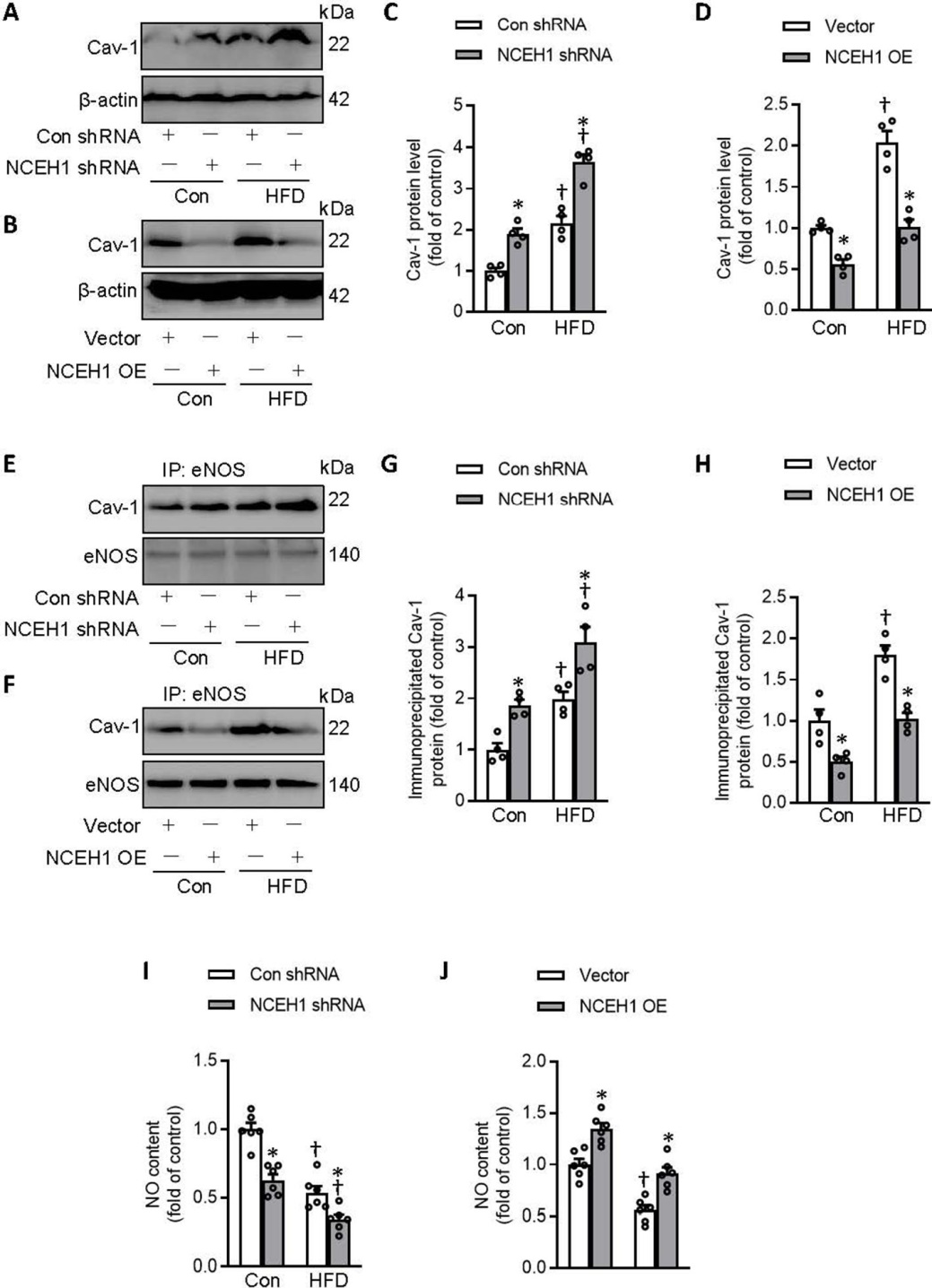
**

**Figure S3. Effects of NCEH1 on Cav-1/eNOS signalling in HFD mice.** (**A,C**) Effects of NCEH1 shRNA on the protein expression of Cav-1 in normal diet- and HFD-induced mouse aortae. (**B,D**) Effects of NCEH1 overexpression on the protein expression of Cav-1 in normal diet- and HFD-induced mouse aortae. **(E,G**) Effects of NCEH1 shRNA on the Cav-1/eNOS complex in normal diet- and HFD-induced mouse aortae. (**F,H**) Effects of NCEH1 overexpression on the Cav-1/eNOS complex in normal diet- and HFD-induced mouse aortae. **(I**) Effects of NCEH1 shRNA on NO production in normal diet- and HFD-induced mouse aortae. (**J**) Effects of NCEH1 overexpression on NO production in normal diet- and HFD-induced mouse aortae. n=4. *P < 0.05 versus Con shRNA or Vector. †*P* < 0.05 versus Con. Differences between groups were assessed with ANOVA followed by Bonferroni post-hoc test. For immunoblotting assay, the ratio of the grayscale values of the target protein and β-actin in each group was normalized by the average value of the control group. NCEH1, neutral cholesterol ester hydrolase 1; NG, normal glucose; HG, high glucose; OE, overexpression; HFD, high-fat diet; ECs, endothelial cells; NO, nitric oxide; eNOS, endothelial nitric oxide synthase; IP, immunoprecipitation.


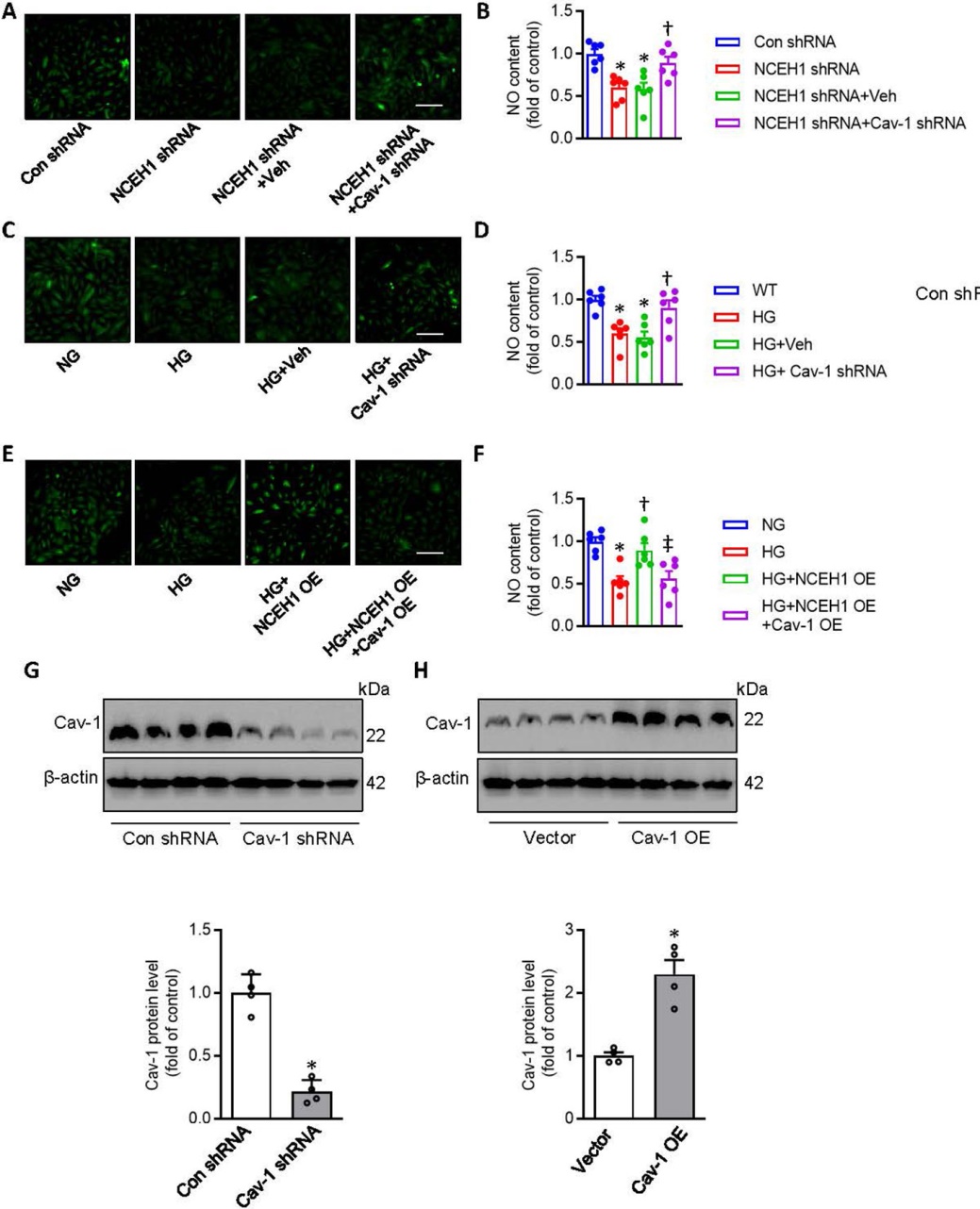


**Figure S4. Effects of Cav-1 on NO production in primary ECs.** (**A,B**) Effects of Cav-1 shRNA on NO production in NCEH1 deficient ECs. Scale Bar, 100 μm. (**C,D**) Effects of Cav-1 shRNA on NO production in HG-exposed ECs. Scale Bar, 100 μm. (**E,F**) Cav-1 overexpression prevented the effects of NCEH1 overexpression on NO production in HG-exposed ECs. Scale Bar, 100 μm. (**G**) Efficiency detection of NCEH1 knockdown in HG-exposed ECs. (**H**) Efficiency detection of NCEH1 overexpression in HG-exposed ECs. n=4. *P < 0.05 versus Con shRNA or Vector. †*P* < 0.05 versus NCEH1 shRNA or HG. ‡P < 0.05 versus HG+NCEH1 OE. The P-value was calculated by unpaired two-tailed Student’s t-test (G,H). Differences between groups were assessed with ANOVA followed by Bonferroni post-hoc test (B,D,F). For immunoblotting assay, the ratio of the grayscale values of the target protein and β-actin in each group was normalized by the average value of the control group. For immunofluorescence staining, the fluorescence intensity value of each group was normalized to the fluorescence intensity average of the control group. Veh, Vehicle; NCEH1, neutral cholesterol ester hydrolase 1; NG, normal glucose; HG, high glucose; Cav-1, caveolin-1; OE, overexpression; ECs, endothelial cells; NO, nitric oxide; eNOS, endothelial nitric oxide synthase.


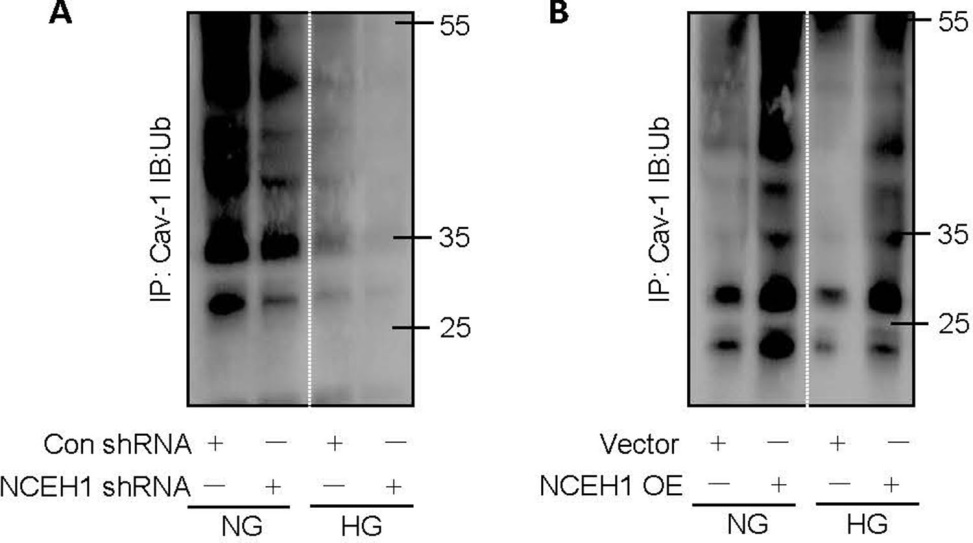


**Figure S5. Detection of Cav-1 ubiquitination in primary ECs.** (**A**) Effects of NCEH1 downregulation on the ubiquitination levels of Cav-1 in HG-exposed ECs. (**B**) Effects of NCEH1 overexpression on the ubiquitination levels of Cav-1 in HG-exposed ECs. NCEH1, neutral cholesterol ester hydrolase 1; NG, normal glucose; HG, high glucose; Cav-1, caveolin-1; OE, overexpression; ECs, endothelial cells. IP, immunoprecipitation.


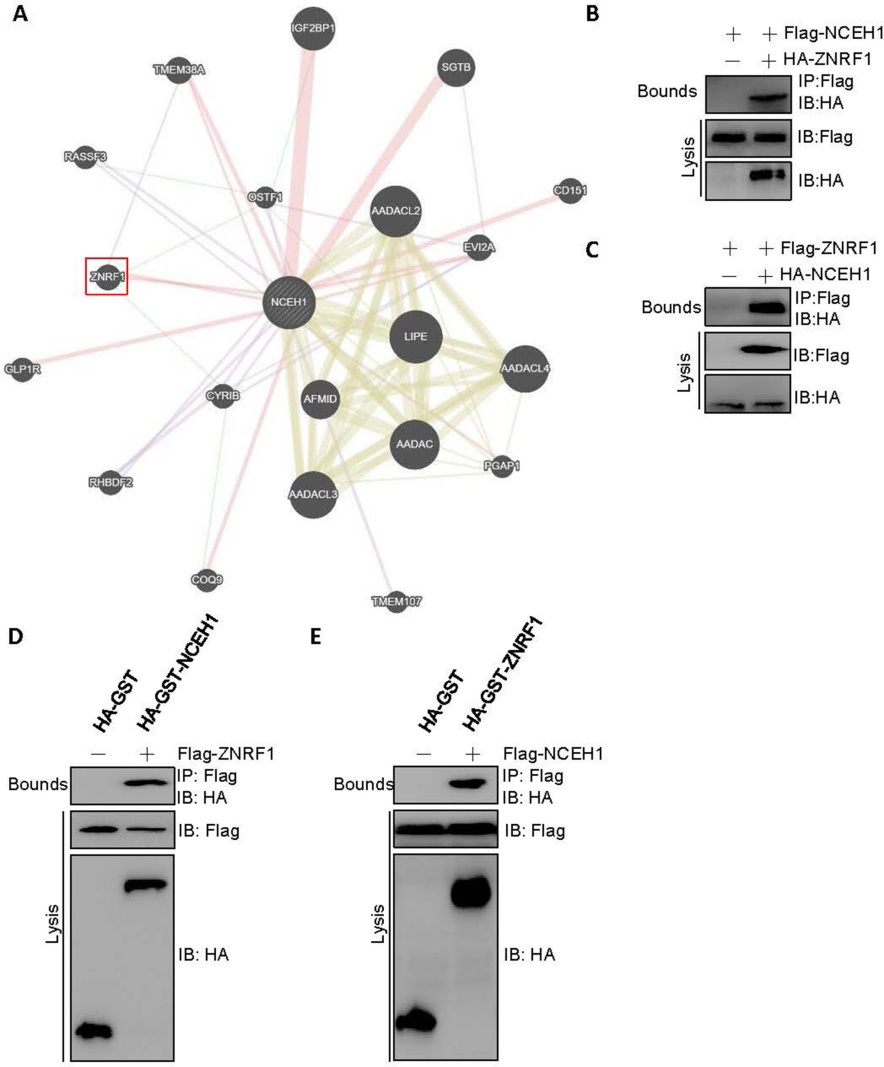


**Figure S6**. **Interaction of NCEH1 with ZNRF1**. (**A**) The protein interaction database Genemania demonstrated the direct interaction of ZNRF1 with NCEH1. (**B**) The co-IP assays in HK293T cells transfected with Flag-tagged NCEH1 and HA-tagged ZNRF1. Anti-Flag and anti-HA antibodies were used as western blot probes. (**C**) The co-IP assays in HK293T cells transfected with Flag-tagged ZNRF1 and HA-tagged NCEH1. Anti-Flag and anti-HA antibodies were used as western blot probes. (**D,E**) GST precipitation assays showing direct ZNRF1-NCEH1 binding. NCEH1, neutral cholesterol ester hydrolase 1; NG, normal glucose; HG, high glucose; Cav-1, caveolin-1; OE, overexpression; ECs, endothelial cells. IP, immunoprecipitation.

**
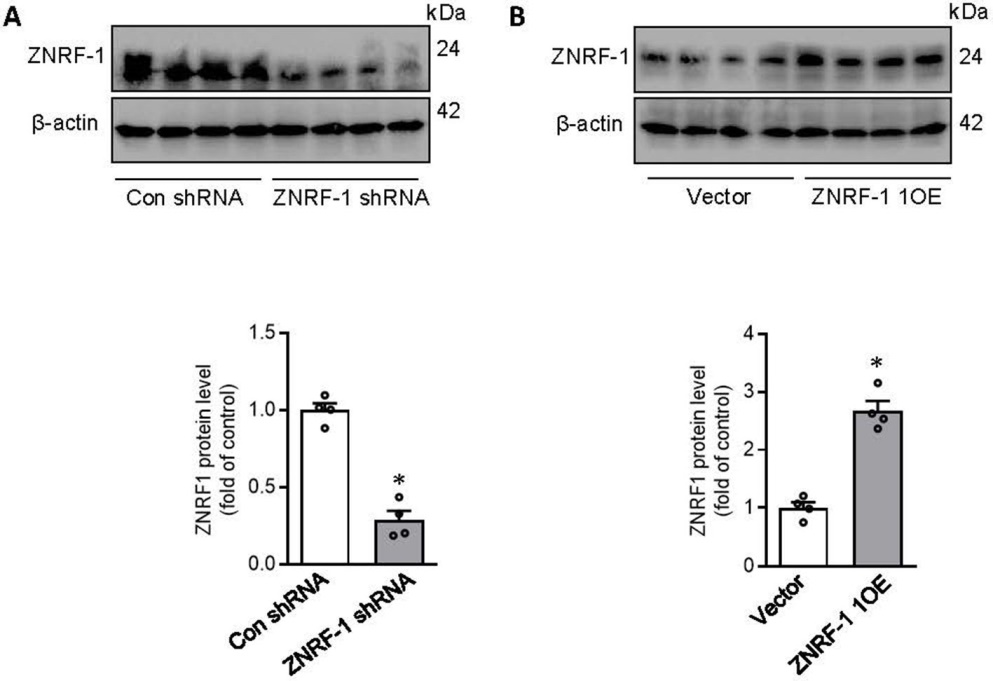
**

**Figure S7. Expression of ZNRF1 in primary ECs.** (**A**) Efficiency detection of ZNRF1 knockdown in HG-exposed ECs. (**B**) Efficiency detection of ZNRF1 overexpression in HG-exposed ECs. n=4. *P < 0.05 versus Con shRNA or Vector. For immunoblotting assay, the ratio of the grayscale values of the target protein and β-actin in each group was normalized by the average value of the control group. HG, high glucose; OE, overexpression.


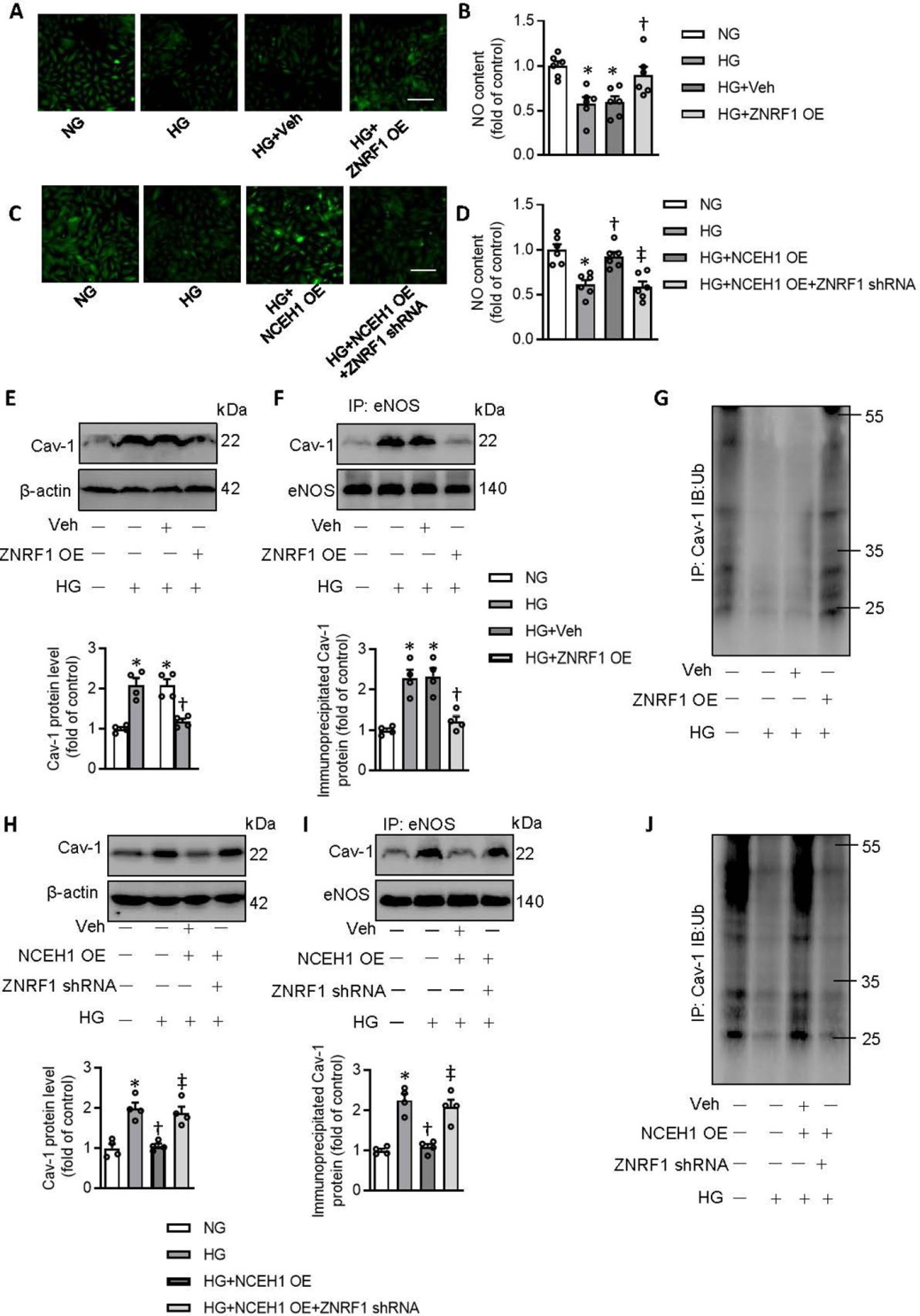


**Figure S8. Effects of ZNRF1 on the Cav-1/eNOS signalling pathway in primary ECs.** (**A,B**) Effects of ZNRF1 overexpression on NO production in HG-exposed ECs. Scale Bar, 100 μm. (**C,D**) ZNRF1 shRNA prevented the effects of NCEH1 overexpression on NO production in HG-exposed ECs. Scale Bar, 100 μm. (**E**) ZNRF1 overexpression prevented the effects of HG on the protein of Cav-1. (**F**) ZNRF1 overexpression prevented the effects of HG on the formation of Cav-1/eNOS complex. (**G**) ZNRF1 overexpression prevented the effects of HG on the ubiquitination of Cav-1. (**H**) ZNRF1 shRNA abolished the effects of NCEH1 overexpression on HG-induced Cav-1 expression. (**I**) ZNRF1 shRNA abolished the effects of NCEH1 overexpression on HG-induced formation of Cav-1/eNOS complex. (**J**) ZNRF1 shRNA abolished the effects of NCEH1 overexpression on HG-induced ubiquitination of Cav-1. n=4-6. *P < 0.05 versus NG. †*P* < 0.05 versus HG. ‡ *P* < 0.05 versus HG+NCEH1 overexpression (OE). Differences between groups were assessed with ANOVA followed by Bonferroni post-hoc test. For immunoblotting assay, the ratio of the grayscale values of the target protein and β-actin in each group was normalized by the average value of the control group. For immunofluorescence staining, the fluorescence intensity value of each group was normalized to the fluorescence intensity average of the control group. Veh, Vehicle; NCEH1, neutral cholesterol ester hydrolase 1; NG, normal glucose; HG, high glucose; OE, overexpression; HFD, high-fat diet; ECs, endothelial cells; NO, nitric oxide; eNOS, endothelial nitric oxide synthase, IP, immunoprecipitation.


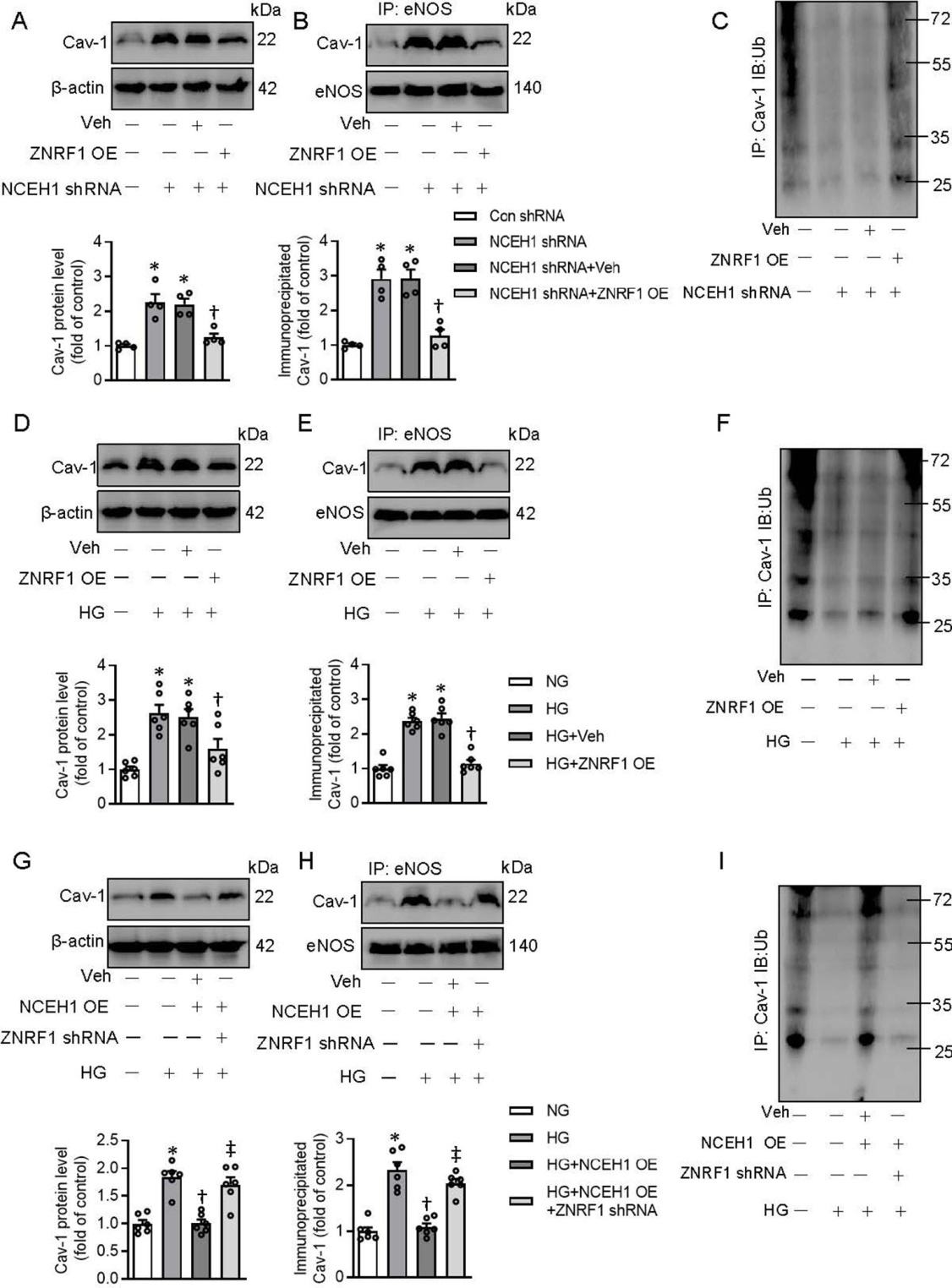


**Figure S9. Effects of ZNRF1 on the Cav-1/eNOS signalling pathway in *ex vivo* mouse aortae.** (**A**) Effects of ZNRF1 overexpression on Cav-1 expression in NCEH1-deficient mouse aortae. (**B**) Effects of ZNRF1 overexpression on Cav-1/eNOS complex in NCEH1-deficient mouse aortae. (**C**) Effects of ZNRF1 overexpression on Cav-1 ubiquitination in NCEH1-deficient mouse aortae. (**D**) Effects of ZNRF1 overexpression on Cav-1 expression in HG-exposed mouse aortae. (**E**) Effects of ZNRF1 overexpression on Cav-1/eNOS complex in HG-exposed mouse aortae. (**F**) Effects of ZNRF1 overexpression on Cav-1 ubiquitination in HG-exposed mouse aortae. (**G**) ZNRF1 shRNA abolished the effects of NCEH1 overexpression on HG-induced Cav-1 expression in *ex vivo* mouse aortae. (**H**) ZNRF1 shRNA abolished the effects of NCEH1 overexpression on HG-induced formation of Cav-1/eNOS complex in *ex vivo* mouse aortae. (**I**) ZNRF1 shRNA abolished the effects of NCEH1 overexpression on HG-induced ubiquitination of Cav-1 in *ex vivo* mouse aortae. n=4-6. *P < 0.05 versus Con shRNA or NG. †*P* < 0.05 versus NCEH1 shRNA or HG. ‡ *P* < 0.05 versus HG+NCEH1 overexpression (OE). Differences between groups were assessed with ANOVA followed by Bonferroni post-hoc test. For immunoblotting assay, the ratio of the grayscale values of the target protein and β-actin in each group was normalized by the average value of the control group. Veh, Vehicle; NCEH1, neutral cholesterol ester hydrolase 1; NG, normal glucose; HG, high glucose; OE, overexpression; NO, nitric oxide; eNOS, endothelial nitric oxide synthase, IP, immunoprecipitation; Cav-1, caveolin-1.


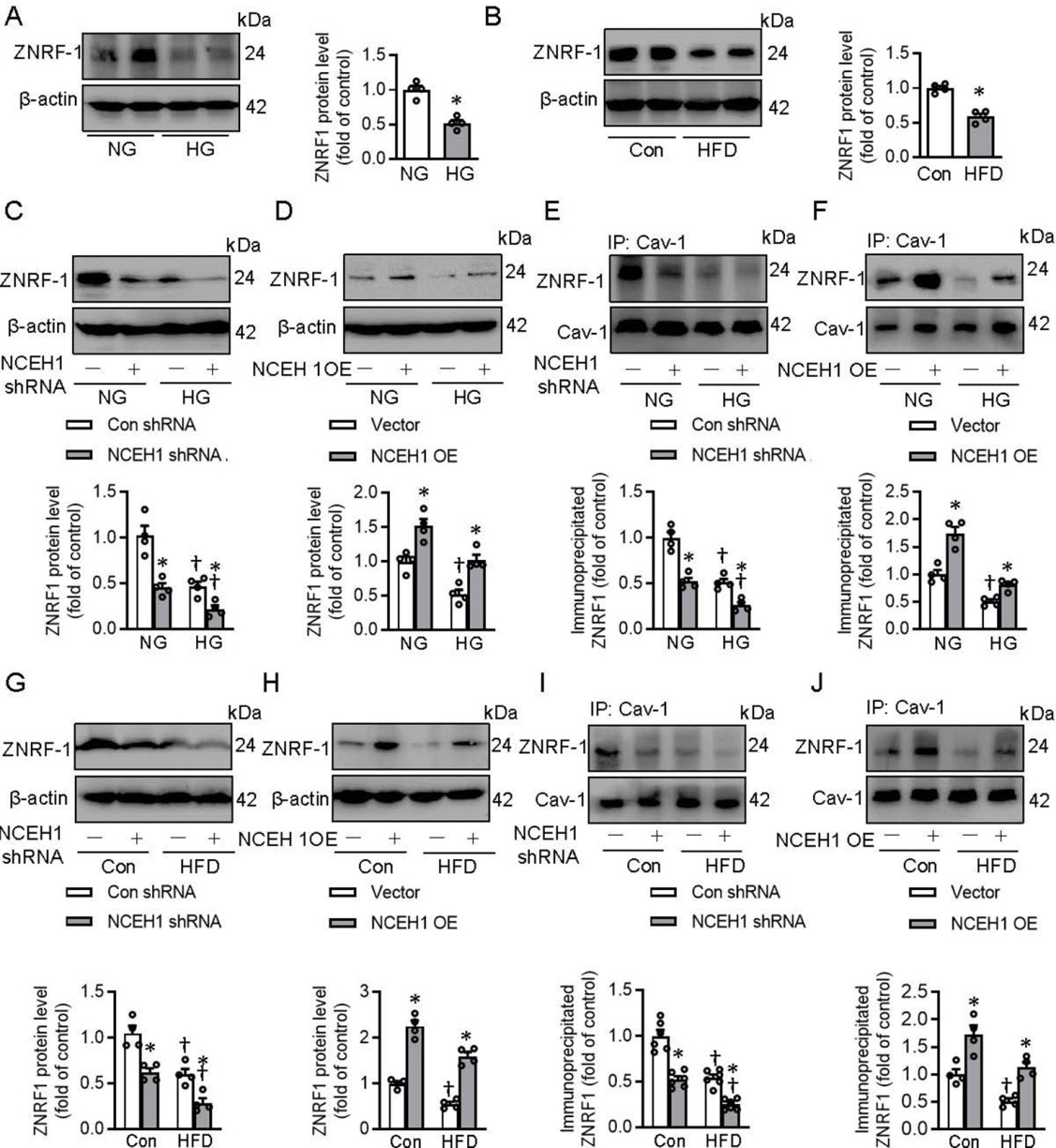


**Figure S10**. **Expression of ZNRF1 in HG-incubated or HFD-induced mouse aortae.** (**A**) The protein expression of ZNRF1 in NG- or HG-exposed mouse aortae. (**B**) The interaction of ZNRF1 with NCEH1 in normal diet- or HFD-induced mouse aortae. (**C**) Effects of NCEH1 shRNA on the protein expression of ZNRF1 in NG- or HG-exposed mouse aortae. (**D**) Effects of NCEH1 overexpression on the protein expression of ZNRF1 in NG- or HG-exposed mouse aortae. (**E**) Effects of NCEH1 shRNA on the interaction of ZNRF1 with Cav-1 in NG- or HG-exposed mouse aortae. (**F**) Effects of NCEH1 overexpression on the interaction of ZNRF1 with Cav-1 in NG- or HG-exposed mouse aortae. (**G**) Effects of NCEH1 shRNA on the protein expression of ZNRF1 in normal diet- or HFD-induced mouse aortae. (**H**) Effects of NCEH1 overexpression on the protein expression of ZNRF1 in normal diet- or HFD-induced mouse aortae. (**I**) Effects of NCEH1 shRNA on the interaction of ZNRF1 with Cav-1 in normal diet- or HFD-induced mouse aortae. (**J**) Effects of NCEH1 overexpression on the interaction of ZNRF1 with Cav-1 in normal diet- or HFD-induced mouse aortae. n=4. *P < 0.05 versus Con shRNA or Vector. †*P* < 0.05 versus NG or Con. The P-value was calculated by unpaired two-tailed Student’s t-test (A-B). Differences between groups were assessed with ANOVA followed by Bonferroni post-hoc test (C-J). For immunoblotting assay, the ratio of the grayscale values of the target protein and β-actin in each group was normalized by the average value of the control group. Veh, Vehicle; NCEH1, neutral cholesterol ester hydrolase 1; NG, normal glucose; HG, high glucose; OE, overexpression; HFD, high-fat diet; ECs, endothelial cells; NO, nitric oxide; eNOS, endothelial nitric oxide synthase; Cav-1, caveolin-1.

**
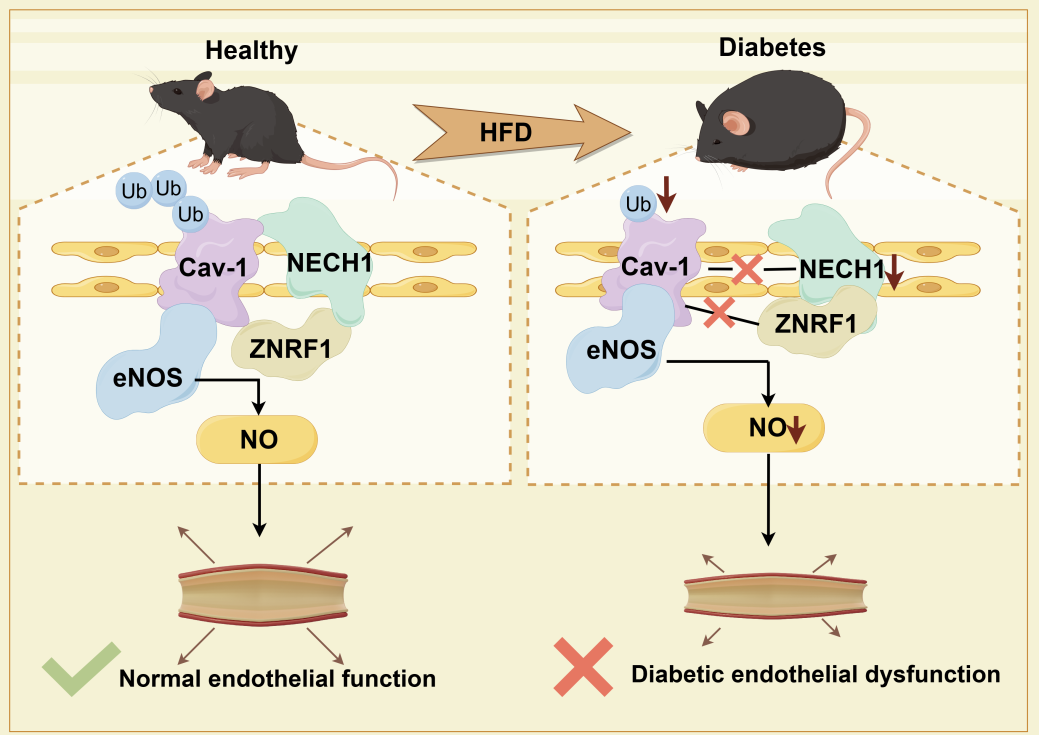
**

**Figure S11**. **Schematic illustration of the proposed molecular mechanism involved in NCEH1-mediated endothelial benefits in obese diabetic mice**.

**Table S1**. **Biochemical characteristics and echocardiographic data of control and T2D mice in the presence or absence of NCEH1**.

| Characteristics | Con shRNA | NCEH1 shRNA | T2D | T2D+NCEH1 shRNA |
| --- | --- | --- | --- | --- |
| Body weight | 29.5±1.2 | 30.2±1.4 | 38.9±1.6* | 39.2±1.3* |
| FBG (mmol/l) | 5.62±0.74 | 5.23±1.01 | 10.29±1.12* | 10.52±1.06* |
| Insulin (ng/ml) | 2.08±0.43 | 2.11±0.39 | 7.61±0.98* | 7.74±1.12* |
| Total cholesterol (mmol/l) | 2.34±0.21 | 2.37±0.22 | 3.44±0.28* | 3.57±0.32* |
| Triacylglycerols (mmol/l) | 1.21±0.11 | 1.22±0.12 | 2.78±0.23* | 2.79±0.22* |

Notes: FBG, fasting blood glucose. n =6. **P* < 0.05 versus Con shRNA, †*P* < 0.05 versus T2D. Differences between groups were assessed with ANOVA followed by Bonferroni post-hoc test.

**Table S2**. **Biochemical characteristics and echocardiographic data of control and T2D mice after overexpression of NCEH1**.

| Characteristics | Vector | NCEH1 | T2D+Vector | T2D+NCEH1 |
| --- | --- | --- | --- | --- |
| Body weight | 30.3±1.1 | 30.1±1.2 | 39.2±1.5* | 39.8±1.4* |
| FBG (mmol/l) | 5.68±0.74 | 5.88±1.02 | 11.11±1.02* | 10.76±0.97* |
| Insulin (ng/ml) | 2.12±0.41 | 2.16±0.32 | 7.77±0.82* | 7.89±1.05* |
| Total cholesterol (mmol/l) | 2.21±0.27 | 2.12±0.24 | 3.55±0.26* | 3.47±0.36* |
| Triacylglycerols (mmol/l) | 1.24±0.12 | 1.27±0.16 | 2.88±0.25* | 2.72±0.24* |

Notes: FBG, fasting blood glucose. n =6. **P* < 0.05 versus Vector, †*P* < 0.05 versus T2D. Differences between groups were assessed with ANOVA followed by Bonferroni post-hoc test.

**Table S3**. **The predicted proteins interacted with NCEH1 using the hitpredict database**.

| **Interactor** | **Name** | **Method Score** | **Interaction Score** | **Confidence** |
| --- | --- | --- | --- | --- |
| P61088 | UBE2N | 0.92 | 0.96 | High |
| P62837 | UB2D2 | 0.88 | 0.937 | High |
| P51668 | UB2D1 | 0.88 | 0.937 | High |
| Q9Y2X8 | UB2D4 | 0.82 | 0.904 | High |
| P51965 | UB2E1 | 0.71 | 0.843 | High |
| P61077 | UB2D3 | 0.67 | 0.815 | High |
| Q96B02 | UBE2W | 0.64 | 0.8 | High |
| P0CG48 | UBC | 0.6 | 0.778 | High |
| P49427 | UB2R1 | 0.5 | 0.71 | High |
| Q13404 | UB2V1 | 0.49 | 0.698 | High |
| Q15819 | UB2V2 | 0.49 | 0.698 | High |
| Q969T4 | UB2E3 | 0.49 | 0.698 | High |
| Q96LR5 | UB2E2 | 0.49 | 0.698 | High |
| O60551 | NMT2 | 0.72 | 0.656 | High |
| **Q8ND25** | **ZNRF1** | **0.42** | **0.647** | **High** |
| Q969U6 | FBXW5 | 0.72 | 0.628 | High |
| P0CG47 | UBB | 0.38 | 0.618 | High |
| Q8NHG8 | ZNRF2 | 0.38 | 0.618 | High |
| Q8NCE0 | SEN2 | 0.62 | 0.585 | High |
| Q6PIU2 | NCEH1 | 0.62 | 0.585 | High |
| O94888 | UBXN7 | 0.61 | 0.58 | High |
| P05023 | AT1A1 | 0.42 | 0.501 | High |
| P27348 | 1433T | 0.38 | 0.479 | High |
| Q6ZTN6 | AN13D | 0.38 | 0.479 | High |
| Q8IZ07 | AN13A | 0.38 | 0.479 | High |
| P50993 | AT1A2 | 0.38 | 0.479 | High |
| P30419 | NMT1 | 0.38 | 0.479 | High |
| Q92731 | ESR2 | 0.38 | 0.458 | High |
| Q93088 | BHMT1 | 0.38 | 0.458 | High |
| P68871 | HBB | 0.38 | 0.458 | High |
| P15104 | GLNA | 0.38 | 0.458 | High |
| P53675 | CLH2 | 0.38 | 0.458 | High |
| Q7Z6J9 | SEN54 | 0.38 | 0.249 | Low |
| Q5JTZ9 | SYAM | 0.38 | 0.249 | Low |
